# Capsaicin Protects Against Cisplatin Ototoxicity by Changing the STAT3/STAT1 Ratio and Activating Cannabinoid (CB2) Receptors in the Cochlea

**DOI:** 10.1038/s41598-019-40425-9

**Published:** 2019-03-11

**Authors:** Puspanjali Bhatta, Asmita Dhukhwa, Kelly Sheehan, Raheem F.H Al Aameri, Vikrant Borse, Sumana Ghosh, Sandeep Sheth, Chaitanya Mamillapalli, Leonard Rybak, Vickram Ramkumar, Debashree Mukherjea

**Affiliations:** Departments of Pharmacology, Internal Medicine and Surgery, SIU School of Medicine, PO Box 19629, Springfield, IL 62794 USA

## Abstract

Capsaicin, the spicy component of hot chili peppers activates the TRPV1 pain receptors, and causes rapid desensitization. Capsaicin also ameliorates cisplatin-induced nephrotoxicity. Cisplatin, a commonly used anti-neoplastic agent for solid tumors causes significant hearing loss, nephrotoxicity and peripheral neuropathy. Upregulation of cochlear TRPV1 expression is related to cisplatin-mediated ototoxicity. Here we report that direct TRPV1 activation by localized trans-tympanic (TT) or oral administration of capsaicin (TRPV1 agonist) prevents cisplatin ototoxicity by sustained increased activation of pro-survival transcription factor signal transducer and activator of transcription (STAT3) in the Wistar rat. Cisplatin treatment produced prolonged activation of pro-apoptotic Ser^727^ p-STAT1 and suppressed Tyr^705^-p-STAT3 for up to 72 h in the rat cochlea. Our data indicate that capsaicin causes a transient STAT1 activation via TRPV1 activation, responsible for the previously reported temporary threshold shift. Additionally, we found that capsaicin increased cannabinoid receptor (CB2) in the cochlea, which leads to pro-survival Tyr^705^-p-STAT3 activation. This tilts the delicate balance of p-STAT3/p-STAT1 towards survival. Furthermore, capsaicin mediated protection is lost when CB2 antagonist AM630 is administered prior to capsaicin treatment. In conclusion, capsaicin otoprotection appears to be mediated by activation of CB2 receptors in the cochlea which are coupled to both STAT1 and STAT3 activation.

## Introduction

Capsaicin is the spicy component of hot chili peppers of the genus *Capsicum* which activates the TRPV1 pain receptors. Capsaicin is a dietary nutraceutical used in cooking spicy Asian food. Absorption of oral capsaicin has been determined to be 94% in the Wistar rat model^1,2^. Capsaicin produces rapid desensitization of TRPV1 receptors which contributes to its use in the treatment of pain in diseases such as arthritis and peripheral neuropathy associated with diabetes^3–6^. Capsaicin is known to possess anti-inflammatory^7^ and anticancer properties^8–10^. Capsaicin has also been shown to ameliorate cisplatin-induced nephrotoxicity^11,12^. Cisplatin chemotherapy is associated with significant hearing loss, nephrotoxicity and peripheral neuropathy. We have previously implicated increased TRPV1 expression in the cochlea in cisplatin-mediated ototoxicity^13^. Other groups have also shown expression and function of TRPV1 in the cochlea^14–16^. Several studies have implicated TRPV1 in mediating entry of cisplatin and aminoglycosides into auditory hair cells^13,16,17^. Local administration of capsaicin by trans-tympanic injection produced temporary hearing loss^18^ which was associated with transient activation of signal transducer and activator of transcription 1 (STAT1)^19^. In contrast, cisplatin produced prolonged activation of Ser^727^ p-STAT1 lasting up to at least 72 h in the rat cochlea following drug administration. Knockdown of STAT1 by siRNA reduced cisplatin ototoxicity^19^, implicating this pathway in cisplatin and possibly TRPV1-mediated hearing loss. The transient nature of the capsaicin-induced hearing loss suggests that it could serve as a preconditioning stimulus to reduce damage to the cochlea produced by ototoxic drugs, such as cisplatin. The goal of this study was to determine whether capsaicin could protect against cisplatin-induced ototoxicity, and if so, to delineate the mechanism(s) underlying such a response.

For these studies we used both the Wistar rat model for cisplatin ototoxicity and an immortalized Organ of Corti hair cell line, UB/OC-1. In this study we compare the p-STAT3 vs p-STAT1 activation by capsaicin and cisplatin separately and together. Our data suggest that both cisplatin and capsaicin activate TRPV1, and STAT1, but produce different downstream signaling pathways. Capsaicin produces a transient activation of STAT1 phosphorylation compared to a sustained STAT1 up-regulation following cisplatin treatment which leads to inflammation and apoptosis. Capsaicin also activates the pro-survival transcription factor Tyr^705^ p-STAT3, whereas cisplatin decreases STAT3 phosphorylation. Thus, there seems to be a dichotomy in the downstream mechanisms activated by capsaicin versus cisplatin in the cochlea. We therefore explored the dichotomy of p-STAT3/p-STAT1 ratio due to capsaicin treatment versus that of cisplatin and discovered that capsaicin increased the p-STAT3/p-STAT1 ratio. This tilted the ratio towards survival. By contrast, cisplatin reversed this ratio leading to cell death. Indeed, pre-treatment with capsaicin prior to cisplatin increases the p-STAT3/p-STAT1 ratio significantly, leading to survival. This led us to investigate other potential upstream targets of capsaicin that activate STAT3.

Interestingly, some endocannabinoids appear to interact with TRPV1 in sensory nerves^20,21^ and since the cochlea is a sensorineural organ, we explored whether capsaicin could activate cannabinoid (CB) receptors in the cochlea. CB2 agonists activate STAT3 and confer protection against oxidative damage in myocardial infarction^2^. Our data indicate that capsaicin indeed increased the expression of cannabinoid receptor CB2 in the cochlea and that leads to the activation of pro-survival Tyr^705^ p-STAT3 transcription factor. The results of this study may have significant translational implications not only for amelioration of cisplatin-induced hearing loss, but also other cochlear inflammatory conditions.

## Results

### Capsaicin protects against cisplatin ototoxicity

**Figure 1:**
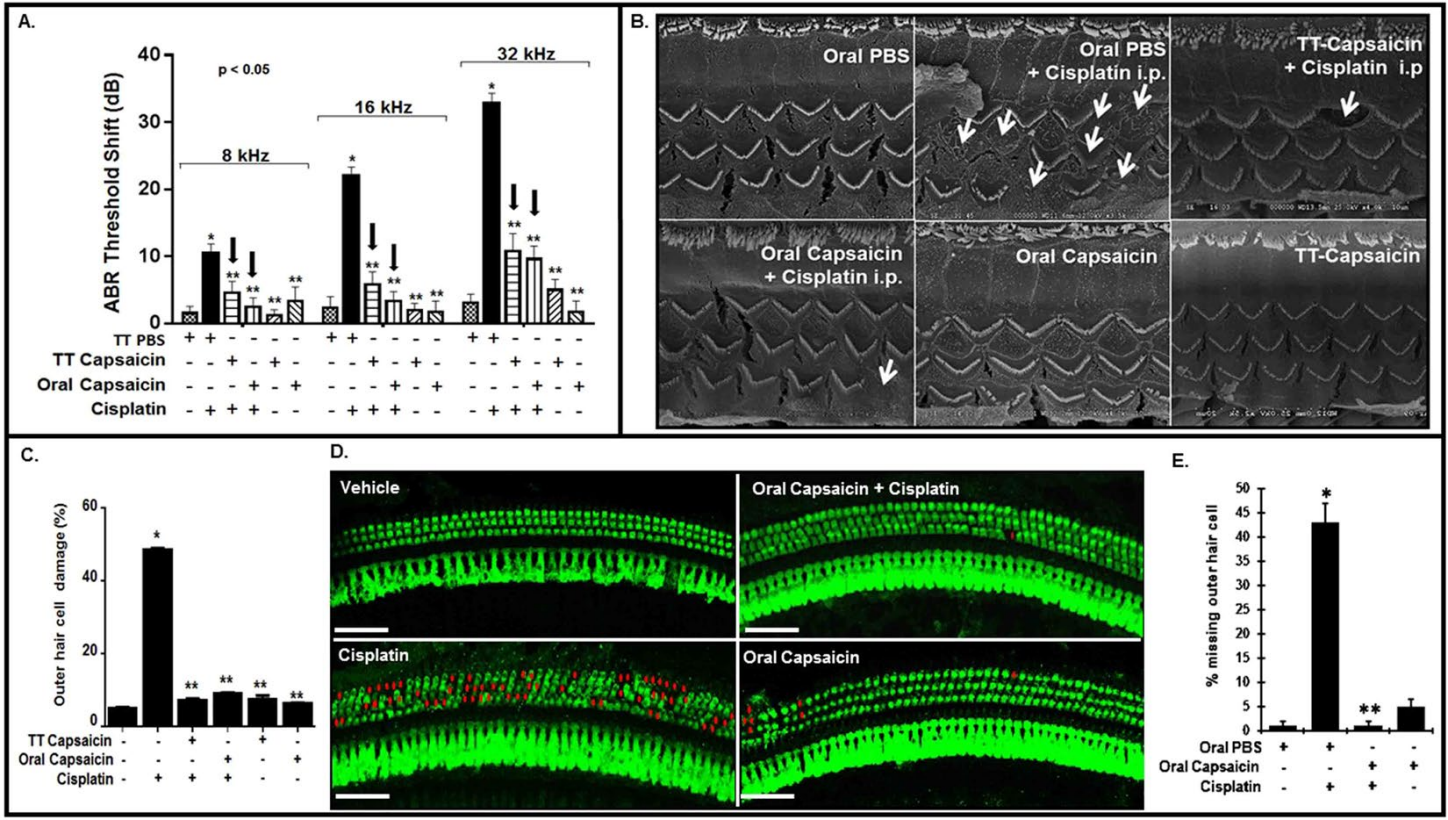
Capsaicin inhibits cisplatin-induced hearing loss. (**A**) We recorded ABR threshold shifts in naïve Wistar rats, pre-treated with either trans-tympanic capsaicin (0.1 µM, 50 µl) or TT-PBS (50 µl), oral PBS or oral capsaicin (20 mg/kg), 24 h prior to cisplatin (11 mg/kg, i.p). We measured post treatment ABR thresholds at 72 h after cisplatin and they showed significant increase with cisplatin alone, which capsaicin attenuated at all frequencies tested. Black arrows indicate significant decrease in threshold shifts when compared to cisplatin. (*Indicates significant difference from control; **indicates significant difference from cisplatin treatment, p < 0.05, n = 9). (**B**) SEM studies performed on rat cochleae show disruption of the stereociliary bundles indicating damage to the OHCs by cisplatin which was significantly ameliorated by pre-treatment with capsaicin. (**C**) Quantitative analysis of outer hair cell damage in the basal turn in the SEM images. (*Indicates significant difference from control; **indicates significant difference from cisplatin treatment, p < 0.05, n = 4). (**D**) Cochlear whole mount preparations from animals stained with myosin VIIA and imaged by confocal microscopy indicate that cisplatin caused hair cell loss in the basal turn (indicated by red arrows), while capsaicin pre-treatment was protective. Scale bar is 25 µm. (**E**) Quantitative analysis of presence or absence of outer hair cells from the basal turn of the whole mount. (*Indicates significant difference from control; **indicates significant difference from cisplatin treatment, p < 0.05, n = 4).

We first assessed ABRs in naïve adult male Wistar rats prior to treatment with either trans-tympanic (TT) vehicle or capsaicin (0.1 µM in 50 µl). Twenty-four hours later, we then infused cisplatin (11 mg/kg) intraperitoneally (i.p) and determined post-treatment ABRs 72 h later to assess hearing loss. Trans-tympanic administration of vehicle (sterile PBS in a volume of 50 µl) produced negligible changes in ABR threshold compared to naïve controls. ABR threshold shifts 72 h following cisplatin administration showed significant elevation. TT-Capsaicin (50 µl of a 0.1 µM solution) pretreatment 24 h prior to cisplatin significantly reduced ABR threshold shifts produced by cisplatin at all three frequencies tested (8, 16 and 32 kHz) (Fig. 1A). TT-Capsaicin administered alone did not significantly alter ABR thresholds, compared to trans-tympanic vehicle-treated rats after 72 h.

Capsaicin is a nutraceutical consumed orally as a pungent spice in several Asian cultures. Therefore, we decided to test the efficacy of oral capsaicin to prevent cisplatin ototoxicity. Oral capsaicin was effective in ameliorating cisplatin-induced hearing loss when we administered it 24 h prior to cisplatin. We observed dose dependent reduction in cisplatin-induced ABR threshold shifts. Capsaicin doses were 5, 10 or 20 mg/kg. We found significant protection in rats administered 10 or 20 mg/kg (Fig. 1A). However, the lowest dose of capsaicin tested (5 mg/kg) did not protect against cisplatin-induced ABR threshold shifts. Capsaicin administered by either trans-tympanic injection or by oral gavage alone did not significantly alter ABR threshold, when assessed 72 h later.

Scanning electron microscopic (SEM) images of the basal turn of the cochlea obtained from the rats treated above showed significant morphological changes in the stereociliary bundles on the outer hair cells (OHCs), indicating damage to the OHCs in the cisplatin group (Fig. 1B). In these studies, cisplatin produced a ten-fold greater loss or damage of outer hair cells compared to vehicle controls. Rats pretreated with trans-tympanic capsaicin had no significant loss of outer hair cells compared to vehicle controls. Oral capsaicin also significantly reduced stereociliary damage induced by cisplatin. Graphical representation of the data is shown in (Fig. 1C). We stained whole mount images of cochleae from similar groups of rats with a polyclonal antibody against myosin VIIa, a hair cell-specific marker. These images supported the SEM findings, wherein cisplatin-induced hair cell loss was significantly decreased by capsaicin pre-treatment (Fig. 1D, red arrows indicative of missing OHCs). Quantitative analyses of hair cell loss indicates that cisplatin-induced outer hair cell death was 43 ± 4%; however, rats pretreated with oral capsaicin prior to cisplatin demonstrated no significant outer hair cell loss compared to control. Oral capsaicin alone produced minimal hair cell damage (Fig. 1E). Capsaicin appears to alleviate cisplatin-induced hearing loss and OHC damage whether administered locally or orally.

### Capsaicin reduces cochlear oxidative stress and inflammatory genes induced by cisplatin

**Figure 2:**
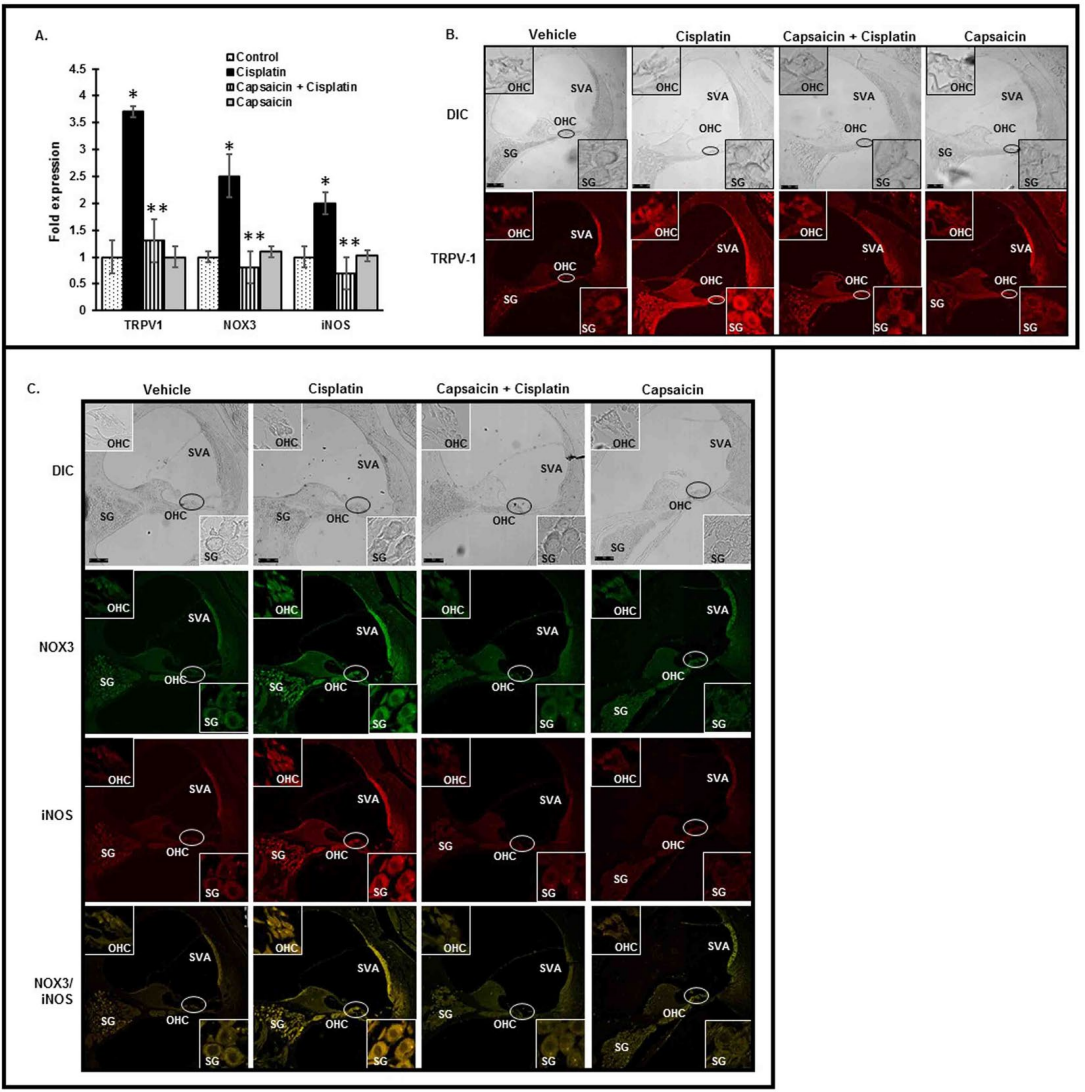
Capsaicin inhibits cisplatin-induced stress and inflammation in the cochlea. We pretreated male Wistar rats with trans-tympanic capsaicin (0.1 µM, 50 µl) 24 h prior to cisplatin (11 mg/kg). We collected -the cochleae 72 h post cisplatin, and saved these specimens either in RNA later or fixed with freshly prepared 4% paraformaldehyde, decalcified and processed them for mid-modiolar sections. (**A**) Quantitative q-PCR analyses from total cochlear RNA indicate that cisplatin treatment increased the relative mRNA expression of TRPV1, NOX3 and iNOS, that was inhibited by trans-tympanic pre-treatment with capsaicin. (**B**,**C**) We used mid-modiolar sections for immunofluorescent staining of TRPV1 (**B**), NOX3 and iNOS (**C**). Cisplatin increased TRPV1, NOX3 and iNOS staining in the OC, SL, SV and SG, which with trans-tympanic capsaicin pretreatment blocked. Images shown are 20X and the insets were imaged at 100x by confocal microscopy. Scale bar is 100 µm. Data presented in (**A**) represent the mean ± S.E.M. of cochleae from four animals from each group. Asterisk (*) indicates statistically significant difference from vehicle, while (**) indicates significant difference from the cisplatin group (p < 0.05).

Cisplatin increased the expression of several cochlear oxidative stress and inflammatory genes. These genes regulate different cellular pathways which culminate in the death of OHCs and other cells of the cochlea, leading to hearing loss^18,22–24^. We examined whether capsaicin could regulate genes linked to cochlear inflammation, such as *TRPV1*, *NOX3* and *iNOS*. Real time quantitative PCR studies of the cochleae 72 h following cisplatin administration indicate 2–3 fold increases in *TRPV1*, *NOX3* and *iNOS* expression. Rats pretreated with TT-capsaicin 24 h prior to cisplatin administration demonstrated significant reduction in the expression of these genes to control levels. Capsaicin administration alone did not significantly change the expression of these genes (Fig. 2A). These data suggest an anti-inflammatory role of capsaicin in the cochlea when combined with cisplatin. We further examined gene expression in mid-modiolar sections of the rat cochlea by immunolabelling for TRPV1, NOX3 and iNOS proteins. Cisplatin increased TRPV1, NOX3 and iNOS immunoreactivity in the organ of Corti (OC), spiral ligament (SL), spiral ganglions (SG) and marginal cells of stria vascularis (SVA) compared to control. Capsaicin pre-treatment prevented this increase (Fig. 2B,C). Fluorescent intensity analyses performed using image J software revealed that cisplatin significantly increased TRPV1, NOX3 and iNOS labelling in the OHCs, SG and SVA compared to control. Capsaicin pre-treatment significantly decreased the expression of these proteins induced by cisplatin, while capsaicin treatment alone did not produce significant change compared to control (Table 1). These data provide additional support for an anti-inflammatory role of capsaicin against cisplatin in the cochlea.

**Table 1:** Capsaicin pretreatment decreases cisplatin-induced stress response.

TRPV1	OHC	SG	SVA
Cisplatin	223 ± 10%*	162 ± 7%*	251 ± 12%*
TT-Capsaicin + Cisplatin	110 ± 7%**	130 ± 9%**	80 ± 5%**
Capsaicin	112 ± 8%	100 ± 5%	108 ± 7%
**NOX3**	**OHC**	**SG**	**SVA**
Cisplatin	185 ± 10%*	320 ± 10%*	260 ± 12%*
TT-Capsaicin + Cisplatin	110 ± 4%**	112 ± 10%**	120 ± 7%**
Capsaicin	97 ± 5%	100 ± 7%	100 ± 6%
**iNOS**	**OHC**	**SG**	**SVA**
Cisplatin	248 ± 15%*	240 ± 12%*	220 ± 10%*
TT-Capsaicin + Cisplatin	127 ± 10%**	125 ± 10%**	118 ± 8%**
Capsaicin	95 ± 3%	100 ± 2%	105 ± 5%

Fluorescent intensity analyses of stress response immune reactivity in rat cochlea: Mid-modiolar sections of rat cochlea were probed with TRPV1, NOX3 or iNOS antibodies and imaged using confocal microscopy. Fluorescent intensity analyses of the images were performed using Image J software. Mean gray values for each region were then used to quantify the increase or decrease in % fluorescence intensity of the various regions of the samples compared to control. Fluorescent intensity analyses represented as % increases or decreases compared to baseline control images (p < 0.05, *significant difference compared to control, **significant difference compared to cisplatin).

### Capsaicin treatment changes p-STAT3/p-STAT1 dynamics during cisplatin insult

**Figure 3:**
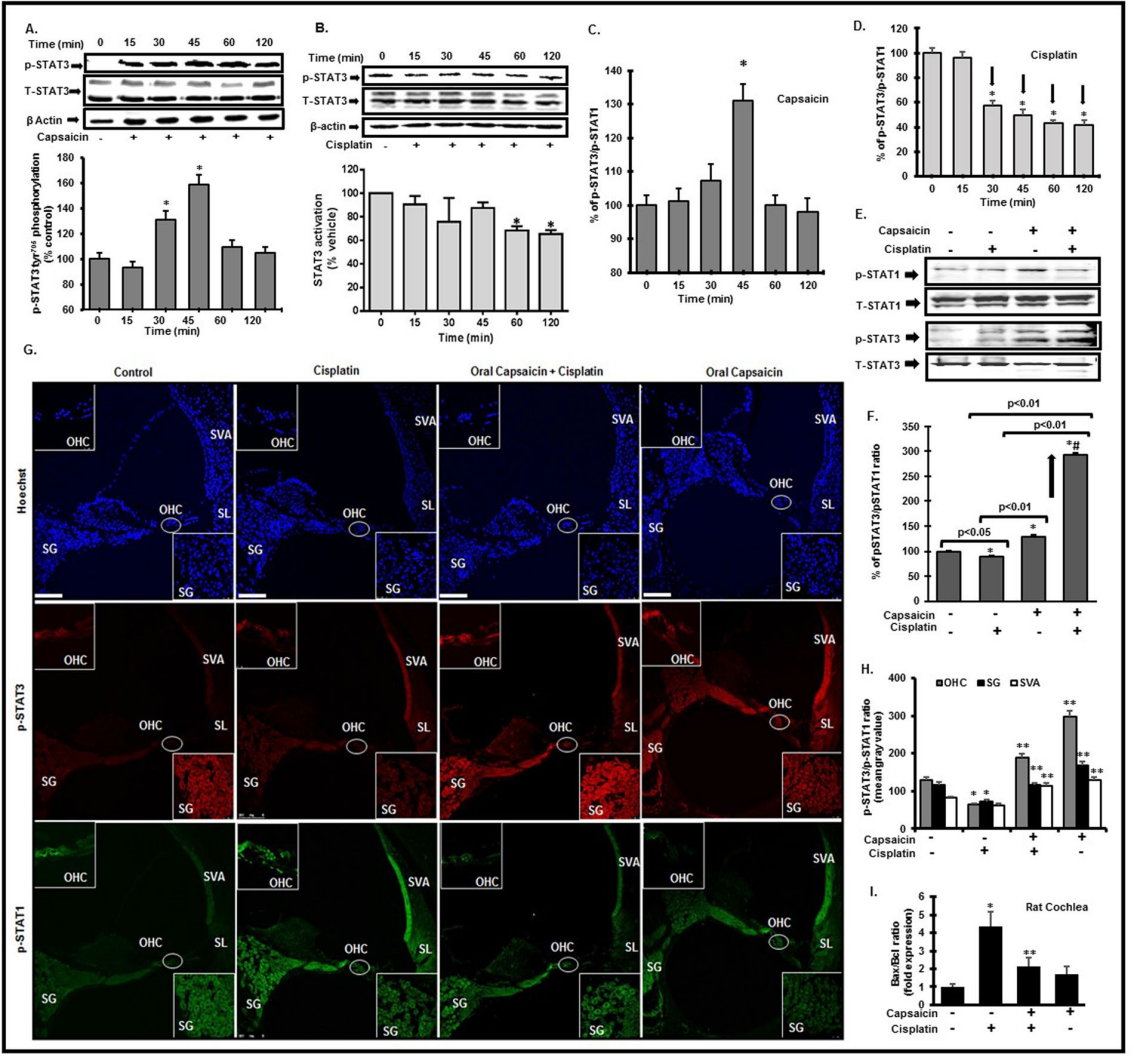
Capsaicin activates both STAT1 and STAT3, while cisplatin activates STAT1 and suppresses pro-survival STAT3. (**A**,**B**) UB/OC1 cells treated with capsaicin (2.5 µM) demonstrated significantly increased Tyr^705^ p-STAT3 after 30 and 45 min (**A**). Cisplatin (2.5 µM) treatment suppressed the Tyr^705^–p-STAT3 (**B**) in a sustained and significant manner at 120 minutes. (**C**) Capsaicin treatment significantly increased the ratio of p-STAT3/p-STAT1 in a transient manner at 45 minutes. (**D**) Cisplatin treatment persistently and significantly decreased the p-STAT3/p-STAT1 ratio. (**E**,**F**) UB/OC1 cells were pretreated with capsaicin (2.5 µM) for 30 minutes followed by cisplatin (2.5 µM) treatment for 45 minutes. Cisplatin increases p-STAT1 significantly, with no change in p-STAT3 phosphorylation, while capsaicin increased p-STAT1 and p-STAT3 significantly. Capsaicin + cisplatin treatment decreased p-STAT1 significantly with a concomitant significant increase in p-STAT3 activation. Graphical analyses of p-STAT3/p-STAT1 ratio indicates that cisplatin significantly decreased the p-STAT3/p-STAT1 ratio compared to control, capsaicin significantly increased p-STAT3/p-STAT1 ratio compared to cisplatin. Capsaicin + cisplatin group showed a significant increase in p-STAT3/p-STAT1 ratio compared to all the groups, possibly due to significant increase in p-STAT3 activation and significant decrease in p-STAT1 activation. (**G**) Mid-modiolar sections of cochleae from rats treated with sterile PBS, i.p. (vehicle), capsaicin (20 mg/kg) or cisplatin (11 mg/Kg, i.p) for 72 hours were immunolabeled for Ser^727^-p-STAT1 (green) or Tyr^705^-p-STAT3 (red) and imaged using confocal microscopy (insets: 100X magnification). Cisplatin increased Ser^727^-p-STAT1 staining at 72 h post administration, while capsaicin treatment did not alter p-STAT1 compared to control in the cochlea. Capsaicin (72 h) increased Tyr^705^-p-STAT3 staining, while cisplatin (72 h) suppressed the Tyr^705^-p-STAT3 staining. Asterisks (*) indicate statistically significant difference from 0 min or control cells (p < 0.05); double asterisks (**) indicate statistically significant difference from cisplatin (p < 0.05); (n = 4). Scale bar is 100 µm. (**H**) Graphical representation of p-STAT3/p-STAT1 ratio of the different cochlear regions as analyzed by image J software. (**I**) Cochlear gene expression by q-PCR indicates that cisplatin treatment increased the Bax/Bcl-2 ratio, which was abrogated by capsaicin pre-treatment.

Our previous study demonstrated that capsaicin stimulated the Ser^727^ phosphorylation of STAT1 which is linked to transient inflammation in the cochlea and temporary hearing loss^18^. We propose that recovery from the transient hearing loss involves resolution of the initial inflammatory response. Capsaicin administration is linked to activation and/or inhibition of STAT3 phosphorylation in cancer cells^8–10^. However, the significance of these findings to cochlear cells under physiological conditions is unclear. We have recently shown that EGCG (epigallocatechin gallate) protects against cisplatin-induced apoptotic cell death by stabilizing the STAT3/STAT1 ratio^25^. In the present study, we monitored the capsaicin-regulated STAT1 and STAT3 activation. The latter promotes anti-inflammatory responses and cell survival^24,25^. We show that capsaicin (2.5 µM) activates STAT1 Ser^727^ phosphorylation in UB/OC-1 cells in a time-dependent fashion, which peaked at 45 min and returned to baseline by 120 min (Supplementary Fig. 1A). Similarly, capsaicin induced Tyr^705^ p-STAT3 phosphorylation was time-dependent, which peaked at 45 min after drug administration and returned to baseline by 120 min (Fig. 3A). This was associated with increased activation of p-JAK2, the immediate upstream regulator of STAT3 phosphorylation at 45 min (Supplementary Fig. 2A). Capsaicin treatment showed significant increase in p-STAT3/p-STAT1 ratio over time with a peak value (~130%) reached in 45 min, with recovery to baseline by 60 min (Fig. 3C), thus tilting the balance of the cell fate towards pro-survival. The ratio of pSTAT3/pSTAT1 was relatively unchanged beyond that time period.

Cells treated with cisplatin (2.5 µM) showed a persistent increase in Ser^727^ p-STAT1, which was still elevated at 120 min (Supplementary Fig. 1B). On the other hand, we observed that cisplatin produced a sustained inhibition of Tyr^707^ p-STAT3 at 120 minutes (Fig. 3B). The ratio of p-STAT3/p-STAT1 following cisplatin treatment is shown in Fig. 3D. This indicates a significant and persistent reduction in this ratio from 30 min to 120 min. Disruption of p-STAT3/STAT1 balance by inhibiting or knockdown of STAT3 sensitizes UB/OC-1 cells to cisplatin^25^. Suppression of p-STAT3 phosphorylation was associated with persistent and significant reductions in JAK2 phosphorylation at 45 minutes up to 2 hours (Supplementary Fig. 2B). We propose that this dichotomy in regulation of the p-STAT3/p-STAT1 ratio between capsaicin and cisplatin could stimulate different sets of genes which could provide cytoprotection in the cochlea by capsaicin. Indeed, UB/OC1 cells pretreated with capsaicin (2.5 µM) for 30 minutes prior to cisplatin treatment for 45 minutes showed significantly decreased p-STAT1 (63.9 ± 3%) with a concurrent increase in p-STAT3 (186.2 ± 6.6%) activation. Cisplatin treatment increases p-STAT1 (120 ± 2%) significantly, with little or no significant change in p-STAT3 phosphorylation (106.5 ± 2.5%), while capsaicin treatment increased p-STAT1 (135.4 ± 4%) and p-STAT3 (173 ± 4%) significantly (Fig. 3E). The p-STAT3/p-STAT1 ratio of the cisplatin treatment alone (88.74 ± 3.8%) was significantly decreased from control. However, capsaicin treatment alone increased the ratio significantly (128 ± 4.1%) compared to control as well as cisplatin, while capsaicin + cisplatin treatment increased the p-STAT3/p-STAT1 ratio to (292.8 ± 5%) of control (Fig. 3F), confirming our hypothesis that capsaicin protects from cisplatin-induced stress response by altering the p-STAT3/p-STAT1 ratio to cell survival.

Immunohistochemistry studies using mid-modiolar cochlear sections from rats treated with cisplatin for 72 h showed up-regulation of Ser^727^ p-STAT1 in the basal turn. Similar mid-modiolar sections from oral capsaicin-treated rats did not show up-regulation in Ser^727^ p-STAT1 (Fig. 3G), indicating inherent differences between these two drugs. Interestingly, oral capsaicin increased Tyr^705^ p-STAT3 phosphorylation as shown in mid-modiolar cochlear sections at 72 h post drug administration, while cisplatin suppressed STAT3 phosphorylation. Mean intensity analyses of the images with image J software indicate that the p-STAT3/p-STAT1 ratio is decreased significantly by cisplatin treatment in the OHC and SG, while SVA showed decreased ratio compared to oral PBS control sections. Oral capsaicin pretreatment significantly increased this ratio compared to cisplatin treatment, while oral capsaicin alone increased the p-STAT3/p-STAT1 ratio in all the three regions of the organ of Corti significantly, compared to control as well as cisplatin (Fig. 3H). The intensity ratios of p-STAT3/p-STAT1 in the different regions of organ of Corti are presented in Table 2. These findings support our hypotheses that capsaicin protects from cisplatin-induced hearing loss not only by inhibiting the pro-inflammatory pathway but also, by stabilizing the p-STAT3/p-STAT1 ratio and tilting the delicate balance towards cell survival. We next monitored two genes, Bax and Bcl-2, whose expressions are regulated by STAT1^26,27^ and STAT3^28,29^, respectively. In UB/OC1 cells, Bax/Bcl-2 ratios were 1 ± 0.2 for control, 2.9 ± 0.1 for cisplatin, 1.31 ± 0.1 capsaicin + cisplatin, and 1.1 ± 0.2 for capsaicin-treated cells, respectively (graph not shown). Rat cochlear gene expression studies showed similar expression patterns of Bax/Bcl-2 ratio. In rats treated with oral PBS we found the ratio was 1 ± 0.2. Rats treated with cisplatin demonstrated a significant increase in the Bax/Bcl-2 ratio. Rats pre-treated with oral capsaicin prior to cisplatin demonstrated a significant decrease of this ratio. Rats treated with oral capsaicin alone showed a ratio less than half of that observed following cisplatin alone (Fig. 3I).

**Table 2:** Capsaicin pretreatment induces pro-survival p-STAT3/p-STAT1 signal.

p-STAT3/p-STAT1 ratio	OHC	SG	SVA
Oral PBS	130 ± 12%	117.15 ± 10%	81.63 ± 5%
Cisplatin	64.1 ± 4%*	72.9 ± 5%*	62.91 ± 5%
Oral-Capsaicin + Cisplatin	189.4 ± 12%**	114.5 ± 8%**	115.62 ± 8%**
Oral Capsaicin	297.4 ± 15%*/**	168.8 ± 11%*/**	130.4 ± 9%*/**

Fluorescent intensity analyses of the pro-survival signal in rat cochlea: Mid-modiolar sections of rat cochlea were probed with p-STAT3 and p-STAT1 antibodies and imaged using confocal microscopy. Fluorescent intensity analyses of the images were performed using Image J software. Mean gray values of p-STAT3 and p-STAT1 were calculated and the ratio for each sample in each group were then calculated as %. (p < 0.05, *significant difference compared to oral PBS group, **significant difference compared to cisplatin treatment group).

### Capsaicin mediated protection is dependent on STAT3 activation

**Figure 4:**
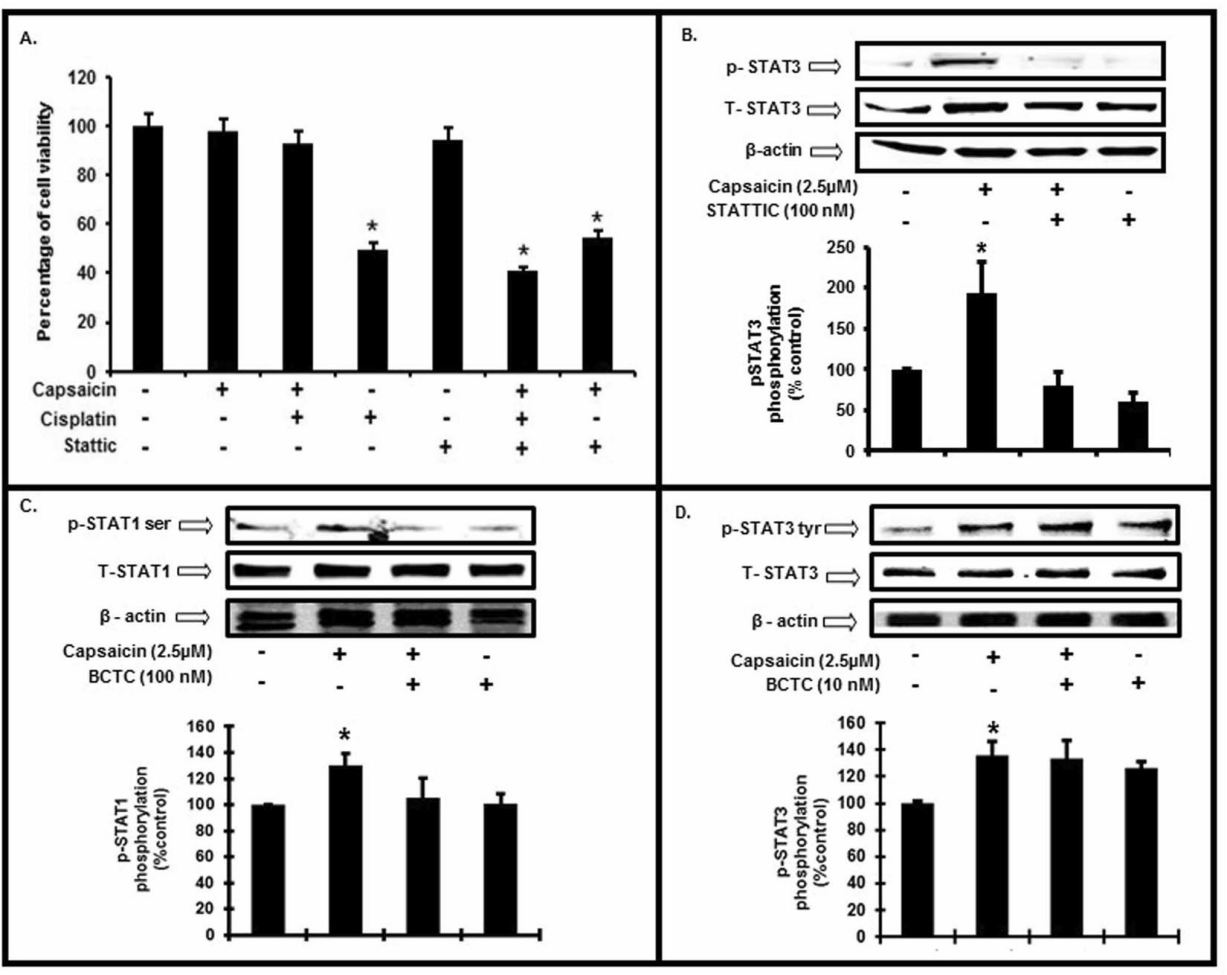
STAT3 is essential for capsaicin mediated protection and is TRPV1 independent. (**A**) We pretreated UB/OC-1 cells with a selective inhibitor of STAT3 (STATTIC, 100 nM) for 45 min, followed by capsaicin (2.5 µM) for 45 min and then cisplatin (20 µM) for 48 h. We assessed the percentage of cell viability by MTS assay. Treatment of STATTIC (100 nM) alone did not induce cell death whereas treatment with cisplatin induced significant cell death. Capsaicin (2.5 µM) protected against cisplatin (20 µM) induced cell death. However, pretreatment with STATTIC (100 nM) (i.e after the inhibition of STAT3), capsaicin could not protect against cisplatin-induced cell death. Asterisk (*) indicate statistically significant difference from control (p < 0.05, n = 8), (**) indicates statistically significant difference from cisplatin (p < 0.05, n = 8). (**B**) We pretreated UB/OC-1 cells with STATTIC (100 nM) for 45 min, followed by capsaicin (2.5 µM) for 45 min. Pretreatment of STATTIC inhibited the phosphorylation of Tyr^705^ p-STAT3. Asterisk (*) indicate significant difference from control (p < 0.05, n = 4). (**C**,**D**) We pretreated UB/OC-1 cells with BCTC (100 nM) for 45 min followed by capsaicin (2.5 µM) for 45 min. Western blots show that inhibition of TRPV1 reduced the Ser^727^ p-STAT1 (**C**) but not Tyr^705^ p-STAT3 (**D**). Western blotting: we loaded all samples serially as depicted on the gel. The grouping of blots, reflects the same blot re-probed with different antibodies. β-actin is used as loading control. None of the blots have lanes taken from different parts of the blot. Thick borders denote separate antibody probes for the same gel.

We next examined the role of the STAT3 pathway in UB/OC-1 cell growth and survival. Capsaicin, added prior to cisplatin, blocked cisplatin-induced cell killing of UB/OC-1 cells. Pre-treatment of UB/OC1 cells with STATTIC (a small molecule inhibitor of STAT3) abrogated the protective action of capsaicin against cisplatin-induced cell killing. Surprisingly, STATTIC also unmasked a toxic action of capsaicin (Fig. 4A). STATTIC (100 nM) significantly reduced the Tyr^705^ p-STAT3 levels (Fig. 4B). This suggests an active role of STAT3 in cytoprotection when cells are challenged with a cytotoxic agent like cisplatin. The cytotoxic effect of capsaicin produced by STAT3 inhibition (see Fig. 4A) implicates this transcription factor in the cell survival/proliferative actions of capsaicin. The lack of effect of STATTIC when added alone would suggest that the STAT3 pathway is not tonically active under normal resting conditions (Fig. 4B).

The importance of STAT3 activation in capsaicin-induced protection of UB/OC-1 cells prompted us to determine whether this is mediated by TRPV1. We tested blockade of the TRPV1 channel by a selective TRPV1 antagonist BCTC (*N-*(4-tertiarybutylphenyl)-4-(3-chloropyridin-2-yl) tetrahydropyrazine-1(2*H*)-carbox-amide, Tocris, Minneapolis, Mn) that has the ability to block the activation of TRPV1 by capsaicin^30,31^. Data shown in Fig. 4C indicate that the use of BCTC (100 nM) decreases the TRPV1 mediated STAT1-Ser^727^ phosphorylation without significantly altering Tyr^705^ STAT3 phosphorylation (Fig. 4D). These data strongly suggest that capsaicin activates STAT1 through a TRPV1-dependent pathway and causes STAT3 phosphorylation independently.

### Capsaicin mediated pSTAT3 activation is cannabinoid receptor CB2 dependent

**Figure 5:**
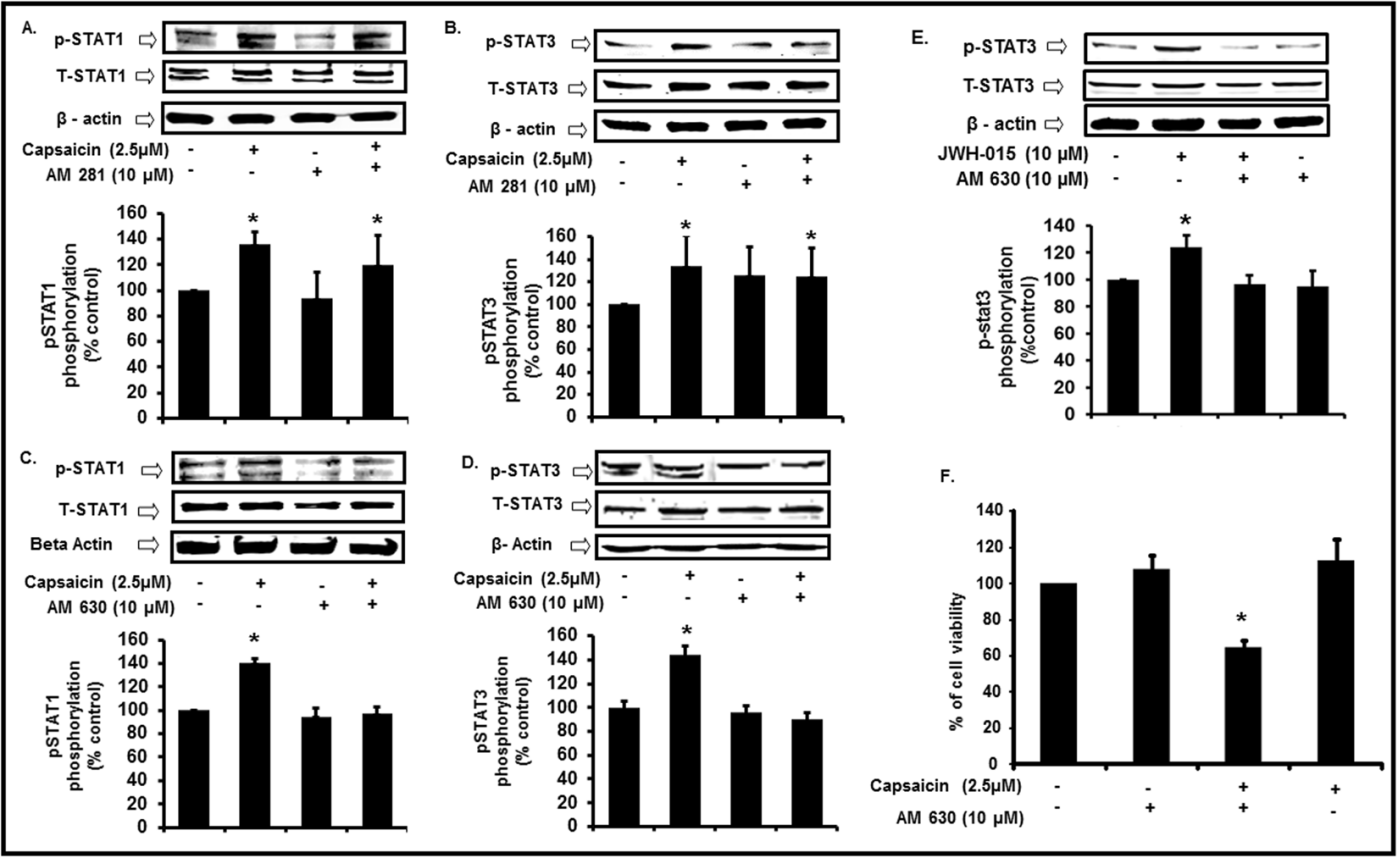
Cannabinoid Receptor CB2 activation is essential for capsaicin induced otoprotection. (**A**,**B**) To determine the role of CB1 receptor in capsaicin induced cell survival, we pretreated UB/OC-1 cells with CB1 antagonist, AM281 (10 µM) for 30 min followed by capsaicin (2.5 µM) for 45 min. Western blots showed that inhibition of CB1 receptors (by AM281) did not affect either the capsaicin-mediated Ser^727^ p-STAT1 (**A**) or Tyr^705^ p-STAT3 (**B**). Asterisk (*) indicates statistically significant change from control. (p < 0.001, n = 4). (**C**,**D**) To determine the role of CB2 receptor in capsaicin induced cell survival, we pretreated UB/OC-1 cells with CB2 antagonist, AM630 (10 µM) for 30 min followed by the capsaicin (2.5 µM) for 45 min. Western blots showed that inhibition of CB 2 receptors blocked the capsaicin-mediated Ser^727^ p-STAT1 (**C**) and Tyr^705^ p-STAT3 (**D**). Asterisk (*) indicates statistically significant change from control. (p < 0.001, n = 4). (**E**) We pretreated UB/OC-1 cells with or without CB2 antagonist, AM630 (10 µM) for 30 min followed by CB2 agonist, JWH-015 (10 µM) for 30 min. Western blots showed CB2 agonist (JWH-015) increased Tyr^705^ p-STAT3, and that inhibition of CB2 receptors (by AM630) reduced the JWH-015 -mediated Tyr^705^ p-STAT3 phosphorylation. Thus, this implicates CB2R in the protective signaling of STAT3. (Asterisk (*) indicates statistically significant change from control (p < 0.05, n = 4). (**F**) We pretreated UB/OC-1 cells with AM630 for 45 min, followed by capsaicin (2.5 µM) for 24 h. We assessed cell viability using MTS assay. Significant cell death occurred when cells were treated with CB2 antagonist, AM630 and capsaicin, implicating CB2 receptors in cell survival. Asterisk (*) indicate statistically significant change from control (p < 0.05, n = 4). **Western blotting:** we loaded all samples serially as depicted on the gel. The grouping of blots reflects the same blot re-probed with different antibodies. β-actin is used as loading control. None of the blots have lanes taken from different parts of the blot. Thick borders denote separate antibody probes for the same gel.

We examined other potential targets of capsaicin to explore whether capsaicin stimulates STAT3 phosphorylation independent of TRPV1. Since some endocannabinoids can interact with TRPV1^20,21^, we reasoned that capsaicin, a classical TRPV1 agonist, could interact with cannabinoid receptors. We treated UB/OC1 cells with either CB1 antagonist AM281 (10 µM) or a CB2 antagonist, AM630 (10 µM) for 30 min prior to capsaicin (2.5 µM). Capsaicin significantly increased p-STAT1 Ser^727^, while AM281 had no effect (Fig. 5A). Likewise, AM281 did not alter capsaicin induced p-STAT3 Tyr^705^ phosphorylation (Fig. 5B). However, inhibition of CB2 receptors with AM630 significantly decreased capsaicin-induced Tyr^705^ p-STAT3 and Ser^727^ p-STAT1. Western blots showed that AM630 pretreatment decreased capsaicin-induced Ser^727^ p-STAT1 phosphorylation (Fig. 5C) and also decreased Tyr^705^ p-STAT3 phosphorylation (Fig. 5D). Moreover, the CB2 receptor agonist JWH-015 increased p-STAT3 and this effect was reversed by AM630 (Fig. 5E). Pre-treatment of UB/OC-1 cells with AM630 30 minutes prior to capsaicin for 24 h significantly decreased cell viability compared to capsaicin (Fig. 5F). Thus, capsaicin likely activated STAT3 via CB2 receptors, which may be essential for cell survival.

### Cannabinoid receptors are expressed in the rat cochlea, and CB2 receptor is essential for capsaicin-induced protection of cisplatin-mediated hearing loss

Previous studies have reported that CB2 receptors are expressed in HEI/OC1 cells^29^ and in the albino rat cochlea^32^. Another study found CB1 expression in SAMP8 mice cochleae^33^. We sought to determine whether cannabinoid receptors (CB1 and CB2) are expressed in UB/OC1 cells and in the rat cochlea. We investigated whether capsaicin treatment could increase the expression of CB receptors. We performed western blotting on UB/OC1 cells treated with capsaicin for 24 h. Capsaicin increased CB2 expression in UB/OC-1 cells (Supplementary Fig. 3A). We determined rat cochlear gene expression levels 72 h after oral capsaicin treatment. Capsaicin increased the expression of CB2 receptor in the rat cochlea ~2 fold relative to basal expression (Supplementary Fig. 3B). We observed CB1 and CB2 receptor expression by immunofluorescent labeling in the organ of Corti in the OHCs, IHC’s, the supporting cells (SC’s), the spiral ganglion and the stria vascularis in mid-modiolar sections of untreated rats. (Supplementary Fig. 3C,D).

**Figure 6:**
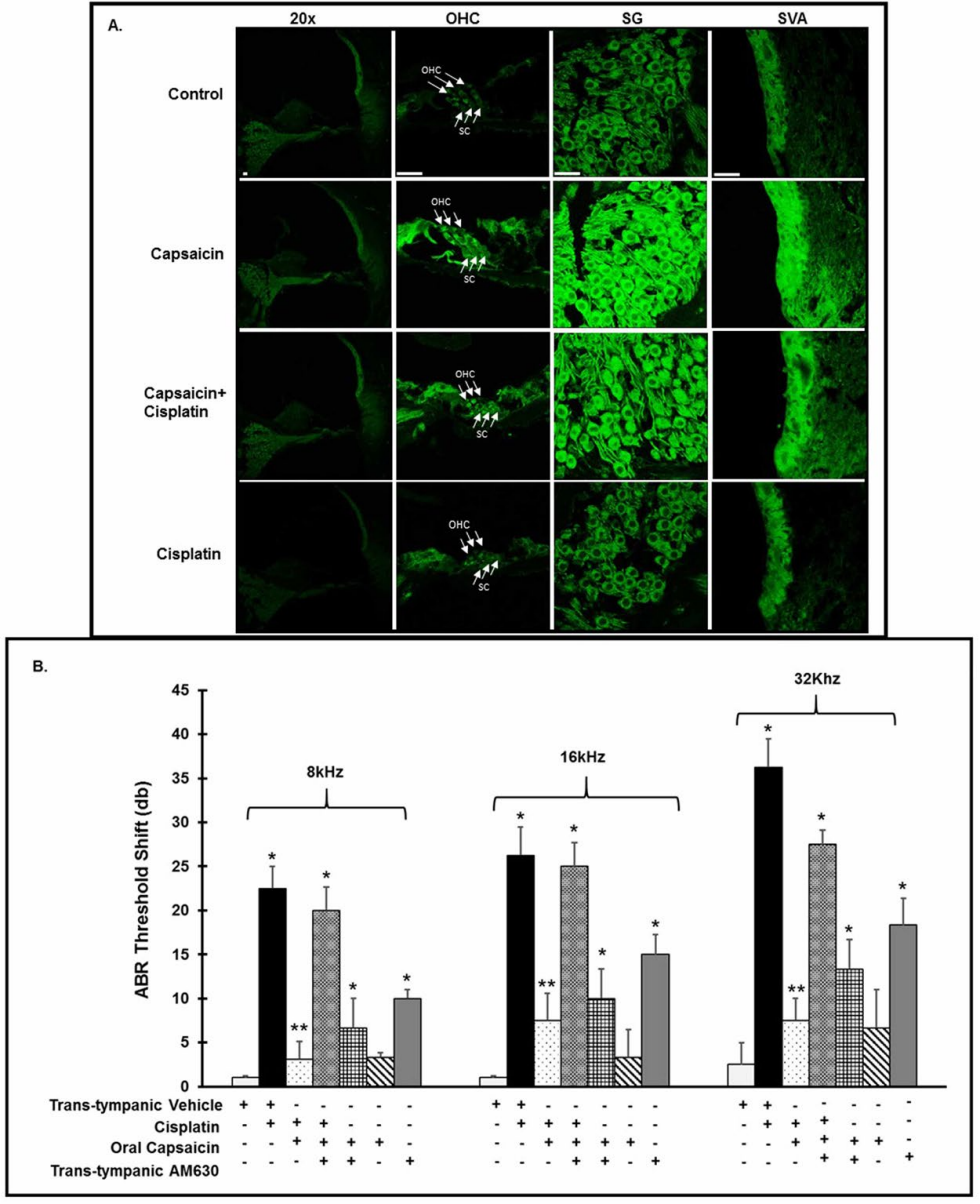
Cannabinoid receptors are expressed in the rat cochlea and CB2 receptor is integral to capsaicin mediated rescue of cisplatin-induced hearing loss. We treated male Wistar rats with oral PBS (1 ml) or oral capsaicin (10 mg/kg) and collected the cochleae 72 h later. (**A**) We immunolabeled mid-modiolar sections of rat cochleae for CB2R and imaged using confocal microscopy at 20X and 100X. We observed CB2R expression in outer hair cells (OHCs), supporting cells (SC’s), spiral ganglion cells (SG) and in the stria vascularis (SVA). Scale bar is 25 µm. (**B**) We pretreated rats were pretreated with AM630 (0.1 µM, TT), CB2R antagonist for 1 hour, then capsaicin (20 mg/kg, 1 ml) by oral gavage 24 hours prior to cisplatin administration (11 mg/kg i.p.). After 72 hours, we determined ABR threshold shifts at 8, 16 and 32 kHz. Pretreatment with capsaicin (20 mg/Kg solution) by oral gavage significantly reduced the threshold shifts caused by cisplatin. CB2 receptor antagonist AM630 (TT) abrogated the capsaicin protection against cisplatin. AM630 (TT) alone also caused ABR threshold shifts. Asterisks (*) and (**) indicate statistically significant increases from PBS and from cisplatin-treated animals, respectively (p < 0.05, n = 4).

We investigated capsaicin induced CB2 receptor activation. We pretreated rats with either vehicle or AM630 by the trans-tympanic route, followed by oral capsaicin (20 mg/kg) or vehicle 4 h later. We administered cisplatin (11 mg/kg, i.p.) 24 h after oral capsaicin and recorded ABR thresholds 72 h post cisplatin. Relative fluorescent intensity (rfi) analyses of mid-modiolar section using confocal microscopy by ImageJ software indicated that cisplatin treatment did not significantly change the level of CB2 labelling in the OHCs, SG’s or in the SVA. However, capsaicin pretreatment significantly increased the expression of CB2 in all the three regions of the cochleae, while capsaicin treatment alone increased CB2 labelling in all the three regions to an even greater extent compared to control (Fig. 6A). Cisplatin produced a 20–40 dB ABR threshold shift with increasing frequencies ranging, from 8–32 kHz, which was abrogated by oral capsaicin. Trans-tympanic administration of CB2 receptor antagonist AM630 reversed the protective effect of oral capsaicin against cisplatin-induced hearing loss. Interestingly, AM630 added either alone or in combination with capsaicin resulted in significant increases in ABR thresholds (Fig. 6B). These latter data suggest that CB2 exerts a tonic protective function in the cochlea and could represent a novel target for otoprotection.

### Capsaicin does not interfere with the antitumor efficacy of cisplatin

**Figure 7:**
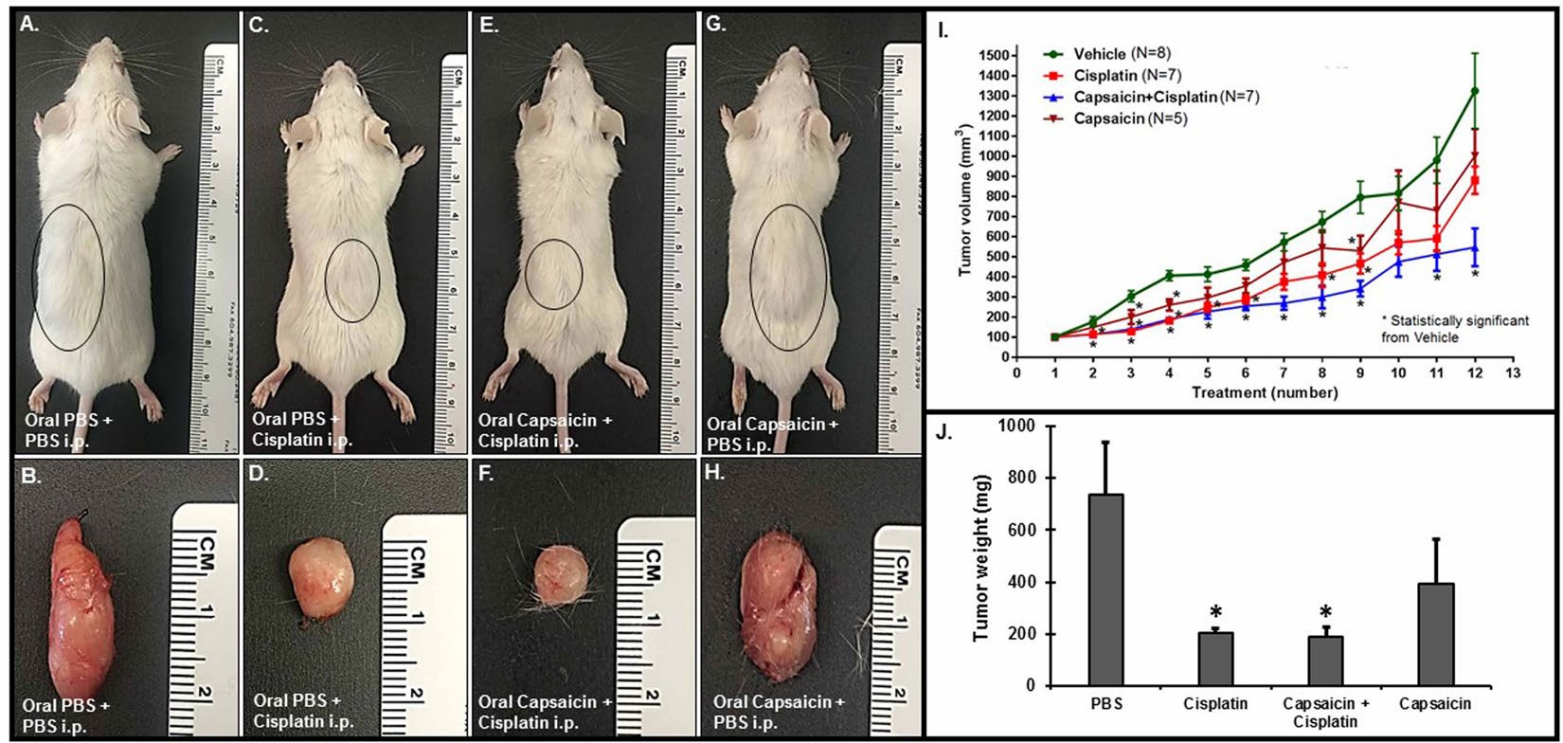
Oral capsaicin pre-treatment does not affect cisplatin’s chemotherapeutic ability in the SCID mouse xenograft model. We injected twenty seven SCID mice with UMSCC-10b cells (1 × 10^6^ cells) subcutaneously and divided them into four treatment groups: Oral PBS + PBS (i.p, 1 ml) (n = 8), cisplatin (2 mg/kg, i.p) (n = 7), oral capsaicin (0.5 mg/kg) + cisplatin (2 mg/kg, i.p) (n = 7) and oral capsaicin alone (n = 5). We administered drugs by oral gavage and/or i.p injections every other day (three times a week, once the tumors were palpable, until the end of the study. We monitored tumor growth closely. Oral capsaicin + Cisplatin and the cisplatin treated mice showed suppressed tumor size (**C**–**F** and **I**) *and weight* (**J**). Capsaicin treatment alone decreased tumor size and weight, but not significantly. We show representative images of the tumor bearing mice and excised tumors at the end of the study from each group in (**A**–**H**). Line graphs and histograms in (**I**,**J**) represent mean ± SEM from each treatment group. Asterisk (*) indicates statistically significant difference (p < 0.05) from vehicle-treated mice.

We employed a SCID mouse xenograft model to determine the effect of oral capsaicin treatment chemotherapeutic efficacy of cisplatin. We injected mice with 1 × 10^6^ head and neck squamous cell carcinoma (UMSCC-10b) cells subcutaneously in the flank region. Tumor growth plotted over a four week period showed a substantial increase in tumor volumes over time which was significantly reduced by cisplatin. Administration of oral capsaicin shifted the cisplatin tumor growth curves to the right, suggesting improved efficacy of this drug combination over cisplatin alone. Tumor weights obtained at the end of the treatment period support these findings (Fig. 7).

## Discussion

**Figure 8:**
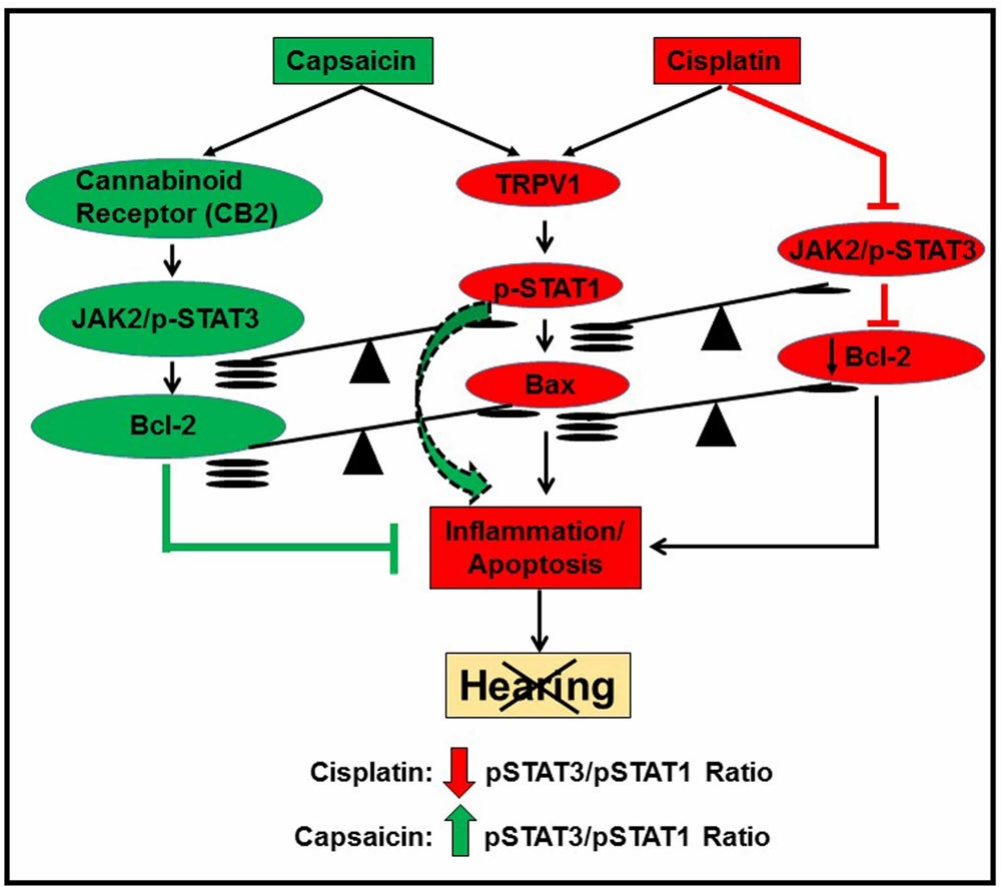
A summary of capsaicin protection against cisplatin ototoxicity. This chart summarizes the proposed molecular mechanisms underlying capsaicin mediated protection against cisplatin-induced ototoxicity. It appears that otoprotection mediated by capsaicin is produced by activation of CB2 receptors which further activates STAT3. Activation of STAT1 by capsaicin contributes to the transient inflammatory response without recruitment of apoptotic pathways as previously observed *in vivo* (as shown by the green dotted arrow). The transient inflammation through the activation of STAT1 desensitizes the TRPV1 receptors, decreasing the presence of STAT1 to be activated by cisplatin. Such phenomenon can mitigate the undesired inflammation further initiated by cisplatin. The net protective action of capsaicin could result from an increase in the JAK2 and STAT3/STAT1 ratio in cells in the cochlea, abrogating the negative impact of cisplatin on this ratio.

The goal of this study was to test the hypothesis that capsaicin could protect against cisplatin-induced ototoxicity and to delineate the mechanisms involved. We developed this hypothesis based on our previous finding that capsaicin produced transient hearing loss in the rat which recovered over a three day period, unlike cisplatin which produced persistent hearing loss^18^. We propose that the transient nature of capsaicin’s action could precondition the cochlea to a subsequent cochlear trauma (i.e. to an ototoxic drug) (Fig. 8). This study confirms the hypothesis that capsaicin could precondition the cochlea and reduce cisplatin ototoxicity. We show that capsaicin, unlike cisplatin, produces transient activation of STAT1 in UB/OC-1 cells and in the cochlea. These findings contrast with the more persistent activation of STAT1 by cisplatin in both UB/OC-1 cells and cochlea and likely result from the more persistent activation of TRPV1 by cisplatin.

Preconditioning utilizes the innate ability of an organ to mount a defense against minor trauma and using that to combat severe trauma. Investigators have utilized this strategy to mitigate pain and nephrotoxicity^7,11,12^. Several groups have studied preconditioning with different agents to prevent noise, age-related or cisplatin-induced hearing loss. Investigators using preconditioning sound in rat and mouse models have demonstrated protection from noise injury as well as age-induced hearing loss^34–36^. Sound preconditioning appears to protect against cisplatin-induced hearing loss^37^. A more recent study suggests that sound preconditioning may work via the induction of heat shock proteins (HSP’s)^38^. A study in zebrafish also showed that dexmedetomidine (DEX) (an alpha-2 adrenoreceptor selective agonist) is effective in preconditioning against cisplatin ototoxicity^39^.

The differences in the ABR threshold shifts in the capsaicin and cisplatin-treated rats were unexpected, given that both capsaicin and cisplatin target TRPV1^13^. Data from our laboratory indicate the involvement of TRPV1 in both capsaicin and cisplatin mediated inflammatory response leading to either a temporary or a permanent ABR threshold shift respectively. Capsaicin mediated TRPV1 activation is transient and resolved within 72 h. Our data further indicate that capsaicin causes an induction of pro-apoptotic STAT1 transcription factor as well as the pro-survival STAT3 transcription factor. This induction of p-STAT3 in the cochlea persists even at 72 h after capsaicin. However, capsaicin transiently induced p-STAT1. This tilts the balance of the p-STAT3/p-STAT1 ratio towards cell survival. Activation of TRPV1 by capsaicin promotes STAT1 activation and the transient induction of inflammatory genes in the cochlea, leading to temporary hearing loss for 24 hr^18^. Similarly, activation of TRPV1 in small diameter C-fiber neurons promotes neurogenic inflammation^4,40^. However, following cisplatin treatment, STAT1 activation persists for 72 h while STAT3 activation is decreased. This alters the balance of p-STAT3/p-STAT1 towards cell death. Cisplatin treatment results in sustained increase in cochlear ROS generation. This leads to TRPV1 activation and persistent inflammation^13^. Thus, TRPV1 appears to be a target for the induction of hearing loss by cisplatin. Knockdown of this protein provided significant protection against cisplatin-induced hearing loss, and apoptosis of outer hair cells^13^. However, TRPV1 agonists appear to be effective in treating bladder pain and over reactive bladder, presumably by inducing desensitization of this receptor^41^. These data suggest a bimodal function of TRPV1 activation by capsaicin but not cisplatin in the cochlea.

The mechanism underlying the transient activation of STAT1 by capsaicin is unclear but may involve rapid de-phosphorylation of this protein by phospho-serine phosphatases, such as protein phosphatase 2A and calcineurin. In addition, it is possible that the increased STAT3 activation produced by capsaicin negatively influences STAT1 activation^42,43^. Another difference between capsaicin and cisplatin is their differing ability to regulate JAK2 and STAT3 phosphorylation. In our cell culture model, we showed rapid phosphorylation of both JAK2 and STAT3 by capsaicin, which persisted for at least 120 min. In contrast, cisplatin produced rapid inhibition of JAK2 and STAT3 phosphorylation. Therefore, the cellular STAT3/STAT1 ratio and cell viability is greater in the capsaicin-treated cells as compared to the cisplatin-treated cells.

We found similar results *in vivo*. Capsaicin increased STAT3 activation without activating STAT1 at 3 days following exposure to cisplatin. In contrast, rats administered cisplatin demonstrated reduced p-STAT3 and greater p-STAT1 in the cochlea than vehicle-treated rats. In nerve injury, STAT3 appears to plav a pro-survival role. This transcription factor appears to be crucial for Schwann cell survival and phenotype conversion for regeneration in mice^44^. STAT3 also inhibits interferon-mediated STAT1 activity and its ability to form homodimers in myeloid cells^45^. Therefore, an increase in STAT3 by capsaicin could serve to diminish the activity of STAT1 and prevent inflammation produced by cisplatin. In contrast, inhibition of STAT3 by STATTIC enhanced killing of UB/OC-1 cells by cisplatin *in vitro*, without affecting survival under basal conditions. Furthermore, STATTIC unmasked a cytotoxic property of capsaicin, likely resulting from unopposed activation of STAT1. STAT3 has important cellular functions linked to cell survival^46^ and the relative proportions of STAT1/STAT3 can regulate the transcriptional activity of anti-apoptotic genes such as Bcl-2 and Bcl-x^27^. Thus, inhibition of STAT3 by cisplatin could play a major role in its initiation of cell death in the cochlea.

Another important finding of this study is that capsaicin-dependent activation of STAT3 is mediated, at least in part, through CB2 receptors. Immunohistochemical studies show expression of CB2 in the cochlea, localized primarily in the OHCs, IHCs, stria vascularis and spiral ganglion cells. Activation of these receptors appears to protect against cisplatin ototoxicity. Inhibition of these receptors by a CB2-selective antagonist reversed this protective action of capsaicin and produced significant hearing loss when added alone. Thus, CB2 receptor activation by endogenous cannabinoids could have a tonic influence on hearing. A recent report^32^ suggests that cisplatin causes a spontaneous increase in CB2 expression in the cochlea. These authors proposed a protective role for CB2 in the cochlea. Cannabinoids appear to exert anti-inflammatory activity in various *in vivo* and *in vitro* models. These agents ameliorate inflammatory conditions^47,48^, presumably via the JAK2/STAT3 pathway. The CB2 agonist HU-308 attenuated cisplatin-induced nephrotoxicity by limiting inflammation, oxidative and nitrosative stress in the kidney^49^.

The otoprotective function of capsaicin did not appear to compromise cisplatin-induced reduction of tumor growth in a SCID mouse xenograft model. In fact, there appeared to be a trend towards increased chemotherapeutic efficacy of combined capsaicin and cisplatin drug compared to cisplatin alone. Therefore, combining oral capsaicin with cisplatin-based chemotherapy could protect against hearing loss without compromising the chemotherapeutic efficacy of cisplatin. The ease of administering capsaicin orally could enhance the feasibility of this approach. Capsaicin is available commercially as a flavored capsule that is easily tolerated. In addition, capsaicin is already in use clinically as a topical ointment for pain and skin conditions, and cannabis derived medicines are also in clinical use for the treatment of various inflammatory diseases. We propose further investigations of capsaicin protection against hearing loss resulting from cisplatin and other inflammatory conditions.

### Proposed mechanism for the protective effect of capsaicin against cisplatin ototoxicity

Capsaicin mediates a transient early inflammatory response in the cochlea. It also activates a delayed anti-inflammatory, pro-survival cascade that protects against cisplatin-induced hearing loss. Capsaicin appears to be otoprotective by desensitization of TRPV1 and also by activation of CB2 receptors. CB2 receptor activation leads to JAK2/STAT3 pro-survival signaling that negates the cisplatin-induced inflammatory/apoptotic (STAT1/Bax:Bcl2) pathways. Capsaicin also activates the TRPV1 pathway transiently which causes an increase in Ca^2+^ release, an increase in ROS generation and increased p-STAT1Ser^727^ phosphorylation. However, apoptotic pathways are not recruited since p53 is not activated^50^. Cisplatin activates TRPV1 receptors chronically. This leads to increases in: Ca^2+^, ROS and p-STAT1Ser^727^ phosphorylation and inflammation. This is followed by recruitment of p53, BAX and caspase cascade^19^. Thus capsaicin shifts the delicate balance of STAT3/STAT1^42^ towards pro-survival signaling, enabling a CB2R activation leading to cell survival. This inhibits damage and apoptosis of outer hair cells and prevents hearing loss in cisplatin treated animals (Fig. 8).

## Experimental Designs, Materials and Methods

### Materials

#### Drugs and reagents

*We* purchased cisplatin, capsaicin, STATTIC, and TRI reagent from Sigma-Aldrich (St. Louis, MO). We obtained CB1 antagonist, AM281, CB2 antagonist AM630, CB2 agonist JWH-015 and BCTC from Tocris biotechne (Minneapolis, Mn). We purchased the CellTiter 96® AQueous One Solution Cell Proliferation Assay from Promega (Madison, WI).

#### Various antibodies used and their dilutions

We obtained CB2 antibody (1:500) from Abcam Inc. (Cambridge, MA). We purchased CB1 (1:500), total STAT1 (1:1000), total STAT3 (1:1000), iNOS, NOX3, and TRPV1 (1:300) from Santa Cruz Biotechnology (Santa Cruz, CA). We bought p-STAT1^Ser 727^ (1:500), p-STAT3^Tyr 705^ (1:500), p-JAK2 (1:1000), from Cell Signaling Technology Inc. (Danvers, MA). We purchased secondary antibodies; goat anti-rabbit, donkey anti-goat and goat anti-mouse from Life Technology (Eugene, OR), and fluorescent tagged (dylight 488 and TRITC) secondary antibodies (1:500) from Jackson Immuno Laboratories (West Grove, PA).

### Cell culture

We obtained UB/OC-1 cells, the immortalized organ of Corti cells derived from the mouse from Dr. Matthew Holley (Institute of Molecular Physiology, Addison Building, Western Bank, Sheffield, UK). We cultured Cells in RPMI 1640 media supplemented with 10% Fetalclone II serum, and penicillin-streptomycin (1 ml/100 ml) and normocin (2 µl/ml). We grew cultures at 33 °C in an incubator with 10% CO_2_. We passaged cells twice a week (1:3-4 media ratio). Dr. Krishna Rao provided to us a head and neck cancer cell line, UMSCC 10B^51^, by Southern Illinois University (SIU) School of Medicine (Springfield, IL, USA). We cultured cancer cells in DMEM (HyClone) supplemented with 10% fetal bovine serum (Atlanta Biologicals Inc., Flowery Branch, GA, USA) and penicillin–streptomycin (Invitrogen).

### Animals

We utilized-adult male Wistar rats (200–350 g) (from Envigo) for this study. We housed the rats in a temperature controlled room with a twelve hour light/dark cycle. They had free access to commercial food and water provided by the SIU Laboratory Animal Care facility. Cisplatin is administered intraperitoneally at 11 mg/kg dose in the rat.

We injected SCID mice (from Envigo, 5–6 weeks of age) subcutaneously with 1.5 × 10^6^ mycoplasma-free UMSCC 10B cells in one flank. When tumors reached palpable size (~100 mm3), attained in 10–15 days after injections, pre-treated mice with oral PBS (vehicle) or oral capsaicin (0.5 mg/kg), and we then treated each group with intraperitoneal PBS or intraperitoneal cisplatin (2 mg/kg). We subsequently treated animals were with oral capsaicin, followed by intraperitoneal cisplatin, on alternate days three times per week for a total of 11 treatments. At the time of each treatment, we calculated tumor volumes based on the formula: volume = width^2^ × (length/2)^52^. We killed mice 24 h after the eleventh treatment. The SIU, Laboratory Animal Care and Use Committee approved and monitored all animal procedures. We confirm that we performed all experiments in accordance with relevant guidelines and regulations of SIU School of Medicine.

### Auditory brainstem evoked responses (ABRs)

Prior to experiments, we anesthetized rats with ketamine HCl /xylazine mixture (24.6/3 mg/kg) and we placed them in a sound-attenuation chamber (Industrial Acoustic Company, Inc.). We maintained body temperature of the rats was at 37 °C with an animal blanket system. We performed pretreatment auditory brainstem responses (ABRs) using an IHS-High-frequency System (Intelligent Hearing Systems, Miami, FL). We exposed the rats to acoustic stimuli via HIS transducers that were directly placed at the entrance of the ear canal. ABR thresholds were obtained for 5 ms duration tone bursts at 8, 16 and 32 kHz at a rate of 50/s. We determined thresholds by visually produced lowest intensity evoked potentials (EP) that progressed in 10 dB steps. We amplified evoked potentials (Eps) to 200,000X,we band pass filtered them (100–3000 Hz) and averaged them over 1024 sweeps^53^.

We performed post treatment ABR’s 72 h following cisplatin administration. At the end of auditory testing, we decapitated the anesthetized animals and harvested the cochlea. The SIU Laboratory Animal Care and Use Committee approved all animal procedures.

### Trans-tympanic injections

We anesthetized male Wistar rats using ketamine/xylazine cocktail. We punctured the tympanic membrane as described in Mukherjea *et al*.^53^.

### Cell Viability Assay (MTS Assay)

We performed the *in vitro* UB/OC1 cell proliferation by using the CellTiter 96® AQueous One Solution Cell Proliferation Assay Kit (Promega, Madison, WI), according to the manufacturer’s instructions. We seeded 3,500 cells per well into a 96-well plate. We pretreated cells were with varying concentrations of capsaicin for 45 mins and cisplatin (20 µM) for 48 h. After 48 h, we added 20 µl of CellTiter 96 AQueous One Solution reagent to each well in 100 µl of total volume of media. We incubated cells for 1 h, and recorded absorbance at 490 nm using an ELISA plate reader. The absorbance is directly proportional to the number of living cells and is expressed as a percent relative to vehicle-treated cells.

### Processing of cochlea for immunohistochemistry

We perfused cochleae with a 4% paraformaldehyde solution, we decalcified cochleae for 7–10 days in 120 mM EDTA, embedded them in paraffin and then sectioned the specimens^53^. We deparaffined slides and rehydrated and stained them with primary and secondary antibodies. We mounted glass coverslips on the specimens with ProLong Diamond mounting medium. We obtained images using a Leica confocal microscope (Buffalo Grove, IL).

### Fluorescent intensity analyses of immunohistochemical images

Image J software was used to analyze the mean gray area for the various images. These values were then used to quantify the increase or decrease in % fluorescence intensity of the various regions of the samples compared to control.

### Morphological studies by scanning electron microscopy (SEM) and Hair cell count

Immediately after completion of post-treatment ABRs, we euthanized rats, harvested their cochleae and processed the specimens as described previously by^54^. We viewed sputter-coated cochlea and photographed them with a Hitachi S-500 scanning electron microscope (Hitachi Ltd.). We examined the resulting scanning electron micrographs to determine qualitative morphological characteristics in various regions including the basal, middle, and apical turns of the rat cochlea.

***Hair cell counts*** were performed as described previously^13^. Two representative areas of the basal turn, middle turn, and apex and hook portion were photographed. In each area, outer hair cells (OHCs) were counted in an area that was 10 pillar cell heads in length. The results are presented as the percentage hair cell damage per cochlear turn. At least three cochleae from different animals per treatment group were used.

### Cochlear whole mount preparation and hair cell count

To study the organization of the outer hair cells and inner hair cells, we used the cochlear whole mount technique. We fixed the cochleae in fresh 4% paraformaldehyde overnight, and then decalcified them in 0.1 mM EDTA for 2 weeks, (pH 7.3) while stirring at room temperature for 2 weeks. After decalcification, we microdissected the cochleae into basal, middle, and apical turns for whole-mount preparation.

For further processing, we washed segments with 1X PBS twice for 5 mins. For blocking, we incubated the segments in 10% blocking serum (horse serum) for 3 hrs at room temperature. We incubated the segments with primary antibody in 0.2% Triton prepared in PBS and refrigerated overnight. The following day, we washed the segments in PBS (three washes) for 5 mins each wash. We incubated the segments in secondary antibody for 2 hrs at room temperature in the dark. We next washed the antibodies with PBS (three washes). We mounted the glass coverslips on the segments with ProLong Diamond Antifade Mountant (Thermo Fisher Scientific) and incubated them at room temperature overnight and then stored them in 4 degrees. We procured the images using a Leica confocal microscope (Buffalo Grove, IL).

#### Hair cell count in whole mount preparations

We selected two to three random areas from the basal turn from each treatment group separately. We counted hair cells in 50 μm and 25 μm segments of the cochlea respectively. Counts were averaged across treatment group. This was further normalized as % of PBS (control) by taking the values of control as 100%.

### RNA isolation

We isolated RNA using RNeasy Mini Kit (Qiagen). We obtained samples from the rat cochleae or UB/OC-1 cell cultures treatments by adding 1 ml TRIZOL reagent to 100 mg of each cochlea or 0.5 ml TRI reagent per well of each six-well plate as further described in Mukherjea *et al*.^13,18^.

### Real-time q-PCR

We converted 500 ng of total RNA to cDNA using iScript cDNA Synthesis Kit (Bio-Rad). We set up the reaction mixture as follows: 4 μl of iScript reaction mix, 1 μl of iScript reverse transcriptase, 1 μg of total RNA and remaining amount of nuclease free water to bring the total volume to 20 μl. We incubated the reaction mix according to the cDNA protocol of our lab (25 °C for 5 min, 42 °C for 30 min and 85 °C for 5 min). We further used the prepared cDNA reaction mix for real-time PCR.

We performed Real-time PCR using Applied Biosystems StepOnePlus machine provided by the Research Imaging Facility (RIF) of SIU. We set up PCR as previously described in detail by^53^. We purchased the primer sets from Sigma Genosys (St. Louis, MO), and these were as follows:

Rodent-Bax (sense): 5′-ATGGCTGGGGAGACACCTGA-3′

(antisense): 5′-GCAAAGTAGAAGAGGGCAACC-3′

Rodent-Bcl2 (sense): 5′-CCTTCTTTGAGTTCGGTG-3′

(antisense): 5′-GAGACAGCCAGGAGAAAT-3′

Rodent-iNOS (sense): 5′-CATTCTACTACTACCAGATC-3′

(antisense): 5′-ATGTGCTTGTCACCACCAG-3′

Rodent-GAPDH (sense): 5′-ATGGTGAAGGTCGGTGTGAAC-3′

(antisense): 5′-TGTAGTTGAGGTCAATGAAGG-3′

Rodent NOX3 (sense): 5′-GTGAACAAGGGAAGGCTCAT-3′

(antisense): 5′-GACCCACAGAAGAACACGC-3′

Rodent-CB2 (sense): 5′-CTCGTACCTGTTCATCAGCAGC-3′

(antisense): 5′-CAGCAGGAAGATAGCGTTGGAG-3′

Rodent TRPV1 (sense): 5′-GGTGGACGAGGTAAACTGGA-3′

(antisense): 5′-GCTGGGTGGCATGTCTATCT-3′

### Western blot analysis

At the end of the capsaicin and cisplatin treatment, we washed UB/OC-1 cells with ice cold 1XPBS with phosphatase inhibitor cocktail added immediately (1:2000 µl) added to it. We prepared total cell lysates using ice-cold lysis buffer containing 50 mM Tris HCl, 10 mM MgCl_2_ and 1 mM EDTA in the presence of protease inhibitors mixture and phosphatase inhibitor 1 (1:100) (Sigma, St. Louis, MO). We kept the lysates on ice for 5 min followed by centrifugation. We transferred the clear supernatant to a clean tube and discarded the pellet. We then mixed the supernatant with 5X solubilization buffer with beta-mercaptoethanol (1:9), and heated on water bath at 95 °C for 6 min.

We then resolved the samples by SDS polyacrylamide gel electrophoresis using 12% gel as described previously by^55^. We then transferred proteins to nitrocellulose membranes, blocked in a 5% BSA solution in TBS (Tris-buffered saline) and refrigerated overnight with the primary antibody in 5% BSA in TBST (Tris-buffered saline, containing 0.15 Tween 20). The following day, after three washes in TBST, we incubated blots with secondary antibody in TBST for 1 h at room temperature. We then washed the blots three times with TBST. We imaged the blot using Li-COR Odyssey, near-infrared imaging system. We performed densitometric analysis of the bands by using Odyssey software. We normalized individual phosphorylated bands to Total ERK, Total JAK2, Total JNK, Total p38, Total STAT1. We used Total STAT3 and β-actin as a loading control. We charted the percentage expression of phosphorylated proteins or other proteins to compare the expression of different treatments.

#### Western blotting

we loaded all samples serially as depicted on the gel. The grouping of blots reflects the same blot reprobed with different antibodies. β-actin is used as loading control. None of the blots have lanes taken from different parts of the blot. Thick borders denote separate antibody probes for the same gel.

### Protein determination

In order to load an equal amount of sample in each well of the gels, we determined the level of protein in samples by the Bradford assay^56^ using bovine serum albumin to prepare standard curves. We calculated the amount of protein to add the exact same amount of proteins for all samples.

### Statistical analysis

We charted data for visual clarification with values presented as mean ± SEM. We performed parametric tests such as Student’s t-test or analysis of variance (ANOVA) followed by Tukey’s post hoc test wherever appropriate in order to determine statistically significant differences among groups. Errors bars shown in the figures represent standard error of mean (SEM).

## Supplementary information

Supplementary Figures
