# Supplementary material for: Capsaicin Protects Against Cisplatin Ototoxicity by Changing the STAT3/STAT1 Ratio and Activating Cannabinoid (CB2) Receptors in the Cochlea
Source: Sci Rep. 2019 Mar 11;9:4131. doi: 10.1038/s41598-019-40425-9 (PMC6411993; doi:10.1038/s41598-019-40425-9)

Capsaicin Protects against Cisplatin Ototoxicity by changing the STAT3/STAT1 ratio and activating Cannabinoid (CB2) Receptors in the Cochlea

Puspanjali Bhatta, Asmita Dhukhwa, Kelly Sheehan, Raheem Alaameri, Sumana Ghosh, Vikrant Borse, Sandeep Sheth, Chaitanya Mamillapalli, Vickram Ramkumar, Leonard Rybak and Debashree Mukherjea

Departments of Pharmacology and Surgery
SIU School of Medicine
PO Box 19629
Springfield, IL 62794

Telephone
217-545-7770

Corresponding author: dmukherjea@siumed.edu

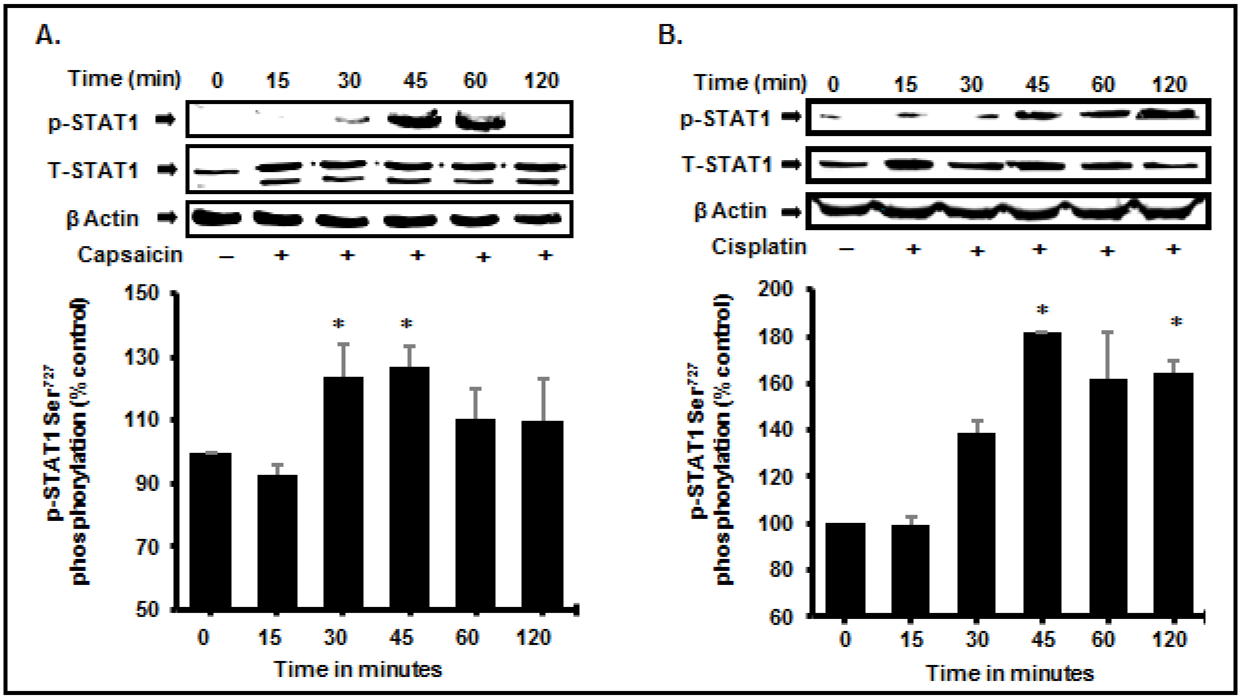

Supplementary Figure 1: p-STAT1 activation by capsaicin and cisplatin in UB/OC1 cells. UB/OC1 cells treated with either capsaicin (2.5 µM) or cisplatin (2.5 µM) and harvested in a time dependent manner. **A.** Capsaicin treatment caused a transient increase of p-STAT1 at 45-60 minutes and reached baseline by 120 minutes. **B.** Cisplatin treatment caused a more prolonged increase lasting till 120 minutes. (Asterisks (*) indicate statistically significant difference from 0 min ($p<0.05$); ($n=4$)).

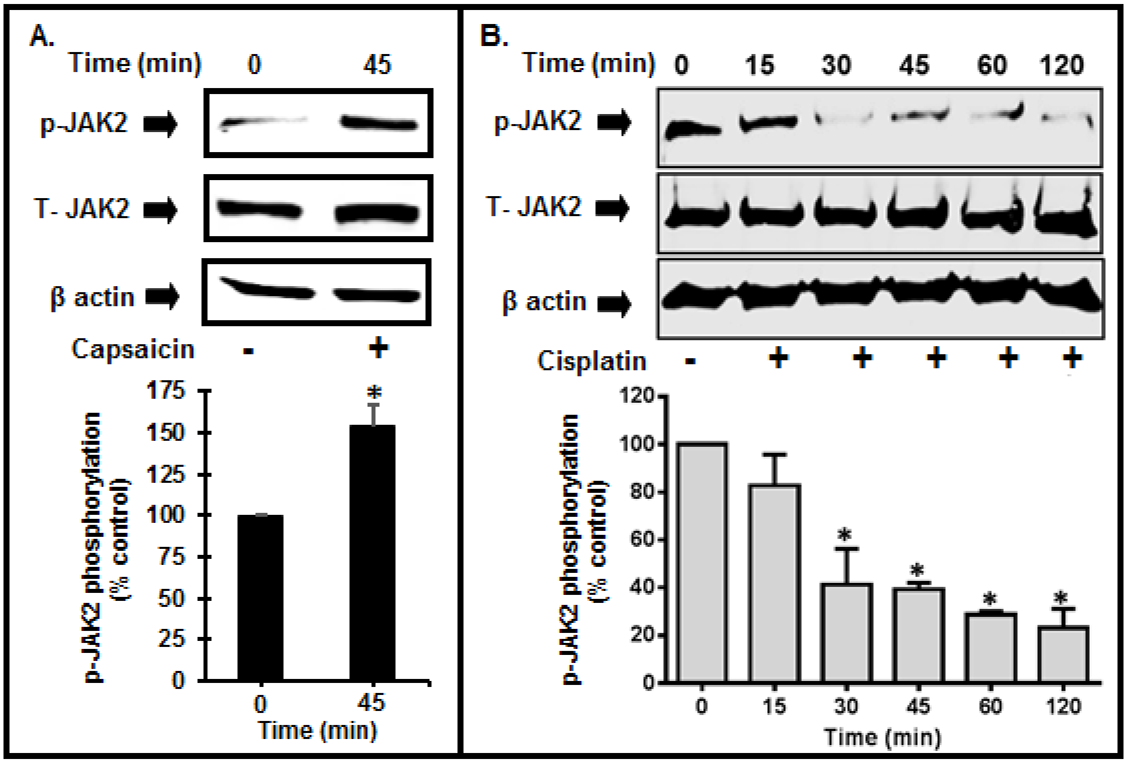

Supplementary Figure 2: Capsaicin activates JAK2, while cisplatin decreases JAK2 phosphorylation. UB/OC1 cells treated with either capsaicin (2.5 µM) or cisplatin (2.5 µM) and harvested in a time dependent manner. **A.** Capsaicin treatment caused a robust increase of JAK2 at 45 minutes. **B.** Cisplatin treatment decreased JAK2 phosphorylation in a sustained manner significantly. (Asterisks (*) indicate statistically significant difference from 0 min ($p<0.05$); ($n=4$)).

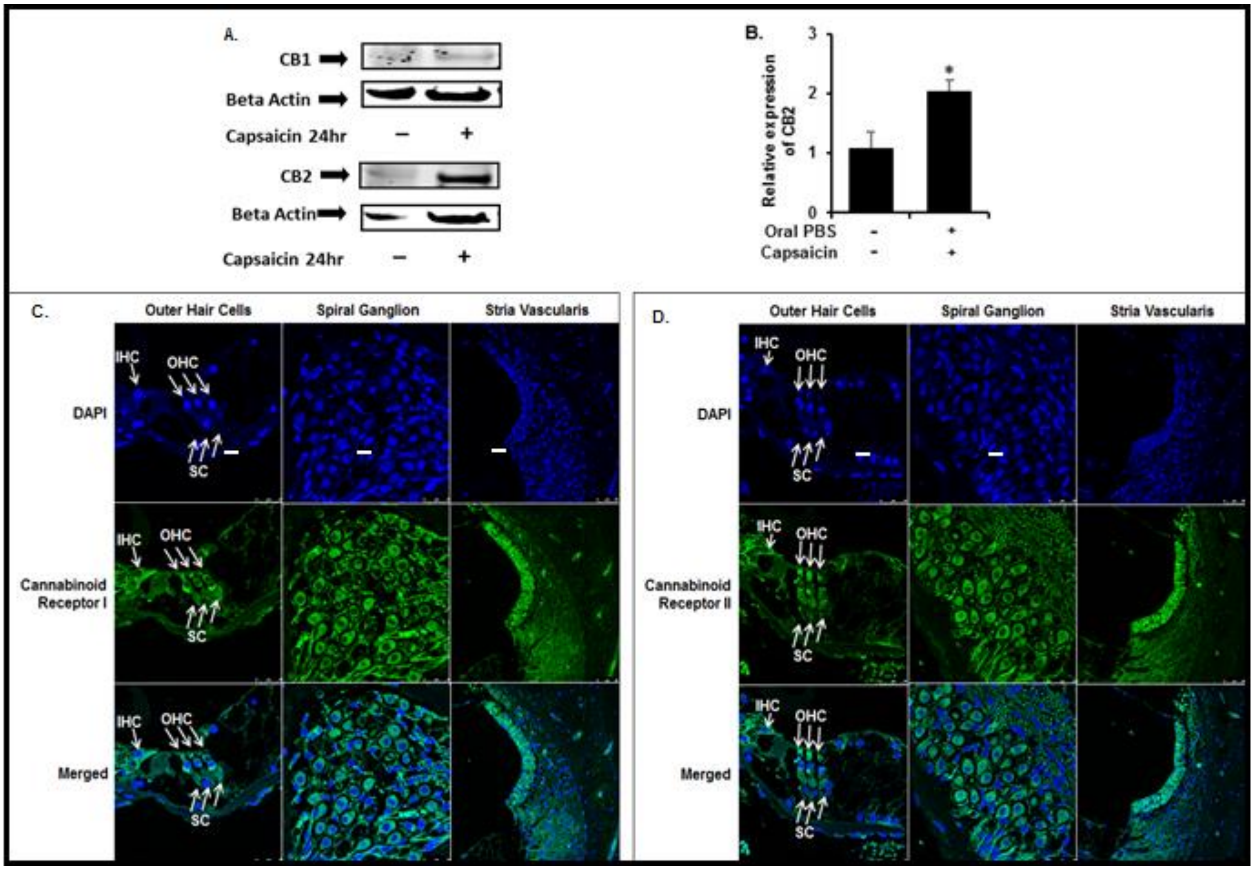

Supplementary Figure 3: Distribution of CB receptors in vitro and in the rat cochlea. A. UBOC-1 cells were treated with capsaicin (2.5 µM) for 24 h. Total protein was extracted and immnoblotting was performed for CB1 and CB2 receptors. B-actin was used for housekeeping and loading control. Capsaicin increased CB2 receptor staining. **B.** Cochleae were harvested from rats treated with either oral PBS or oral capsaicin (20mg/kg) for 24h. Total RNA was harvested, converted to c-DNA and probed for CB2 receptor expression by q-PCR. GAPDH was used as housekeeping gene. Capsaicin treatment shows an increase in CB2 expression in the rat cochlea. **C and D.** Cochleae were harvested from rats treated with PBS, fixed in 4 % paraformaldehyde in PBS for 8 h. They were then decalcified in EDTA (10 mM), and mid modiolar sections of the cochleae were probed for the cannabinoid receptors subtypes, CB1 **(C)** and CB2 **(D)**, while nuclei were stained with DAPI. Fluorescent images were obtained with a Leica confocal microscope. CB1 and CB2 immunoreactivity were seen in the IHC's, OHCs, stria vascularis and spiral ganglion cells of the basal turn of the rat cochlea. Magnification for OHC, spiral ganglion cells and stria vascularis (40 X). Similar images were obtained from 3 different animals.

Capsaicin Protects against Cisplatin Ototoxicity by changing the STAT3/STAT1 ratio and activating Cannabinoid (CB2) Receptors in the Cochlea

Puspanjali Bhatta, Asmita Dhukhwa, Kelly Sheehan, Raheem Alaameri, Sumana Ghosh, Vikrant Borse, Sandeep Sheth, Chaitanya Mamillapalli, Vickram Ramkumar, Leonard Rybak and Debashree Mukherjea

Departments of Pharmacology and Surgery
SIU School of Medicine
PO Box 19629
Springfield, IL 62794

Telephone
217-545-7770

Corresponding author: dmukherjea@siumed.edu

Figure 3A: Capsaicin mediated activation of p-STAT3 time course

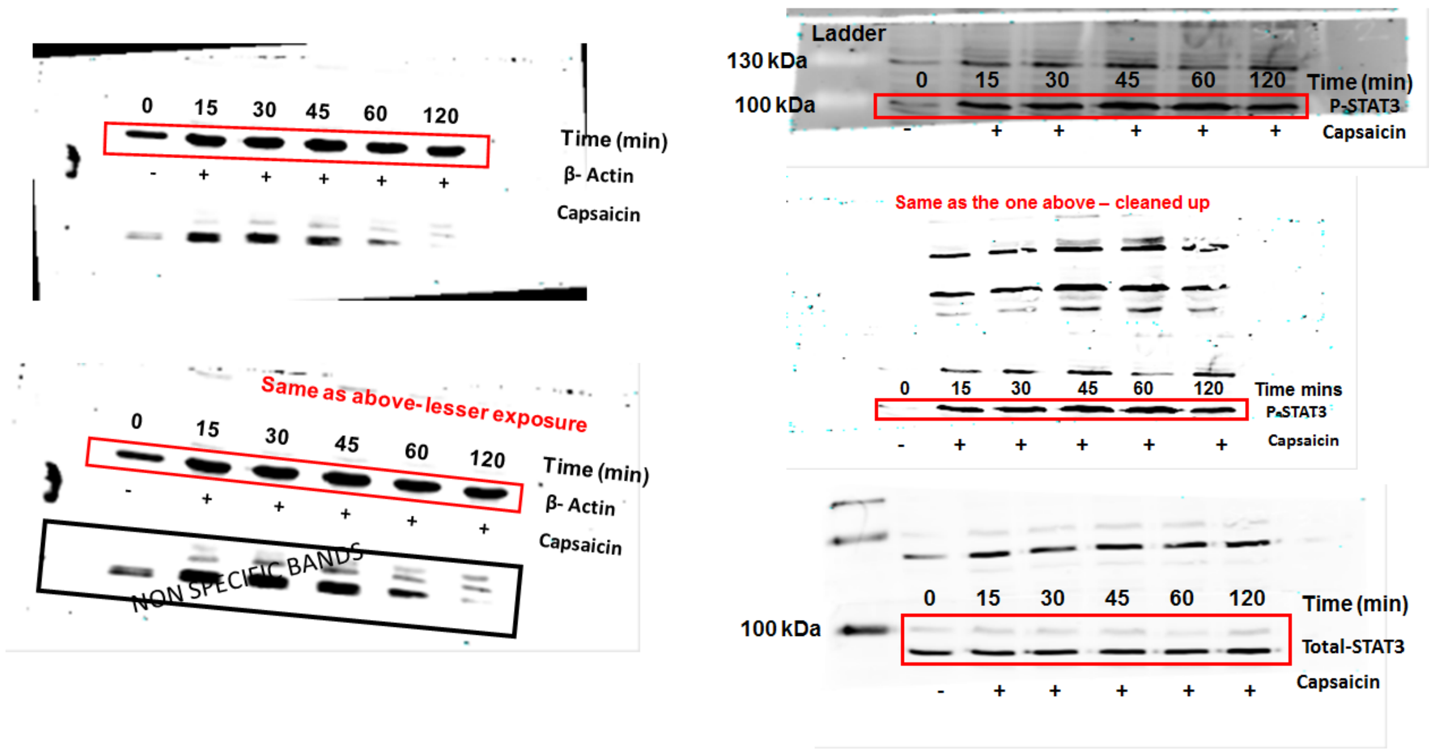

Figure. 3B: Cisplatin decreases p-STAT3: We could not find the original blots for this figure as the files were corrupted. However, we are including another blot of the same experiment showing similar trend/result as that depicted in the figure. Blots on the left are same blots as on the right, just different exposures.

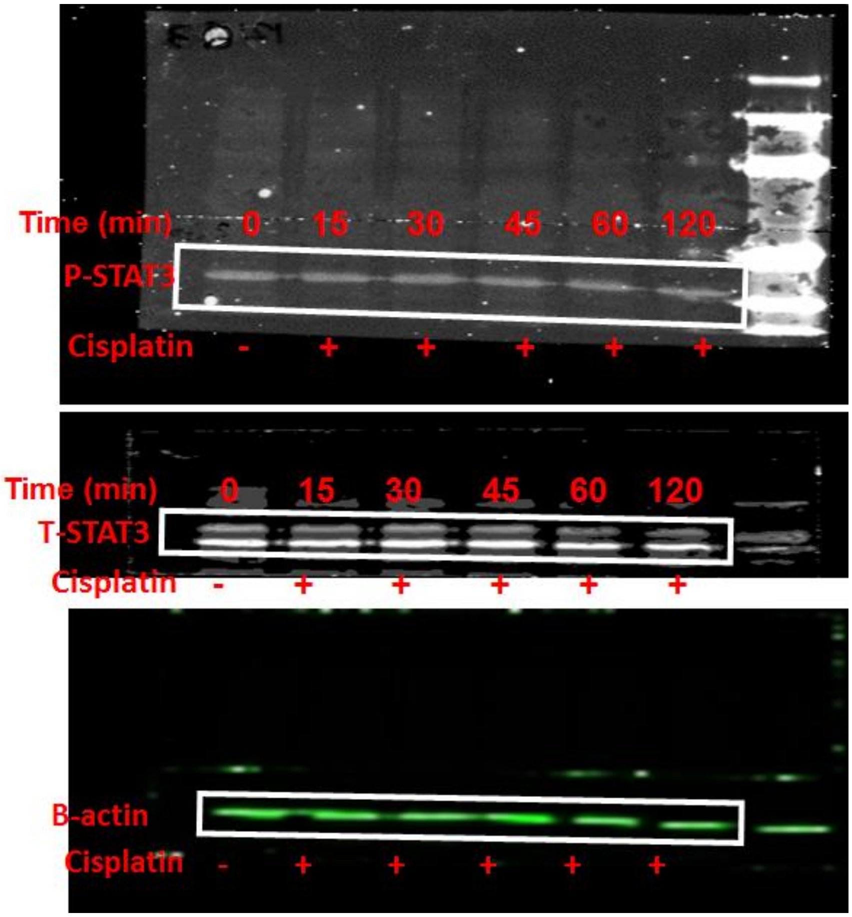

Figure. 3E: Capsaicin + cisplatin decreases p-STAT1 activation and increases p-STAT3 activation.

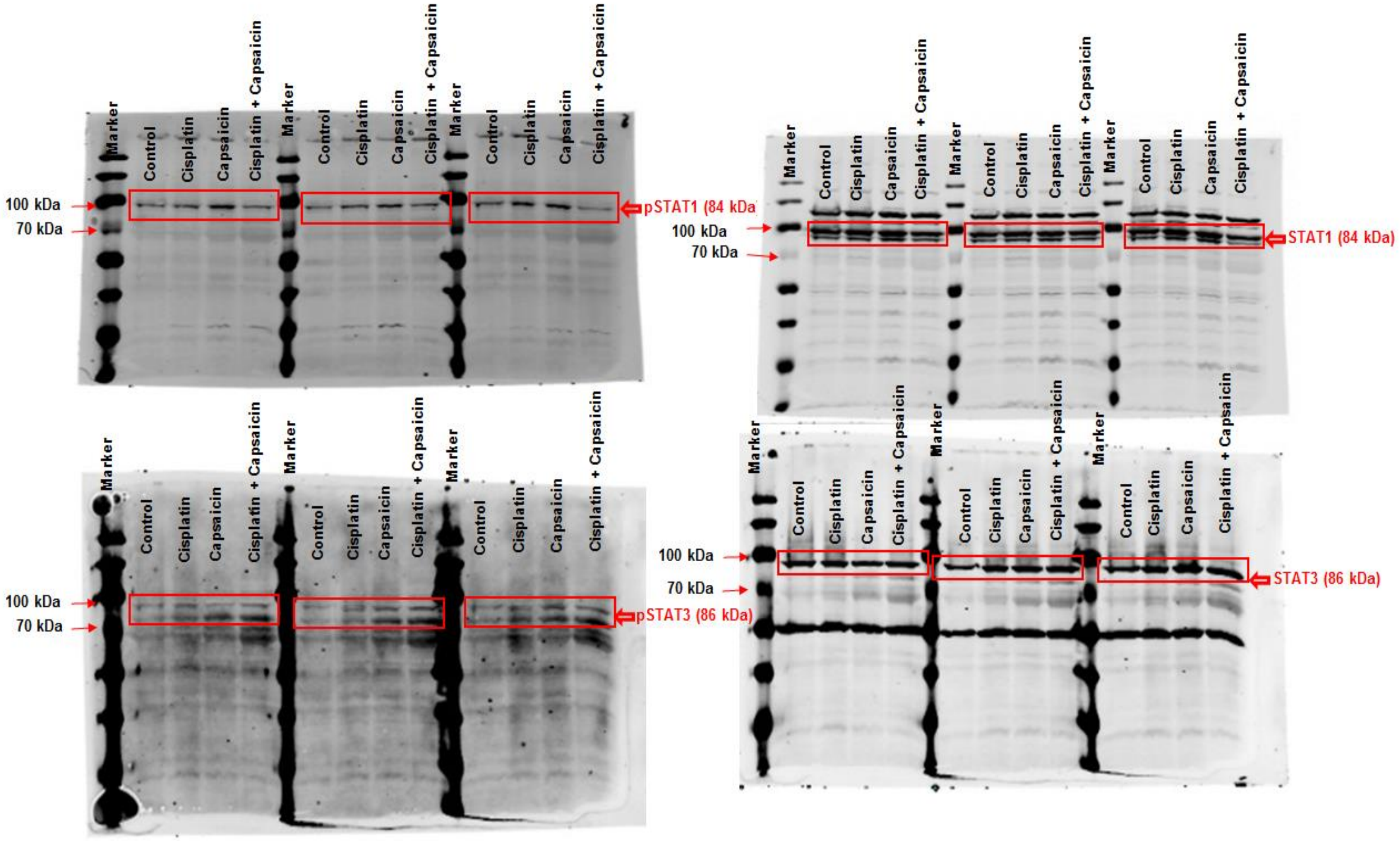

Figure 4B: Capsaicin increases STAT3 phosphorylation.

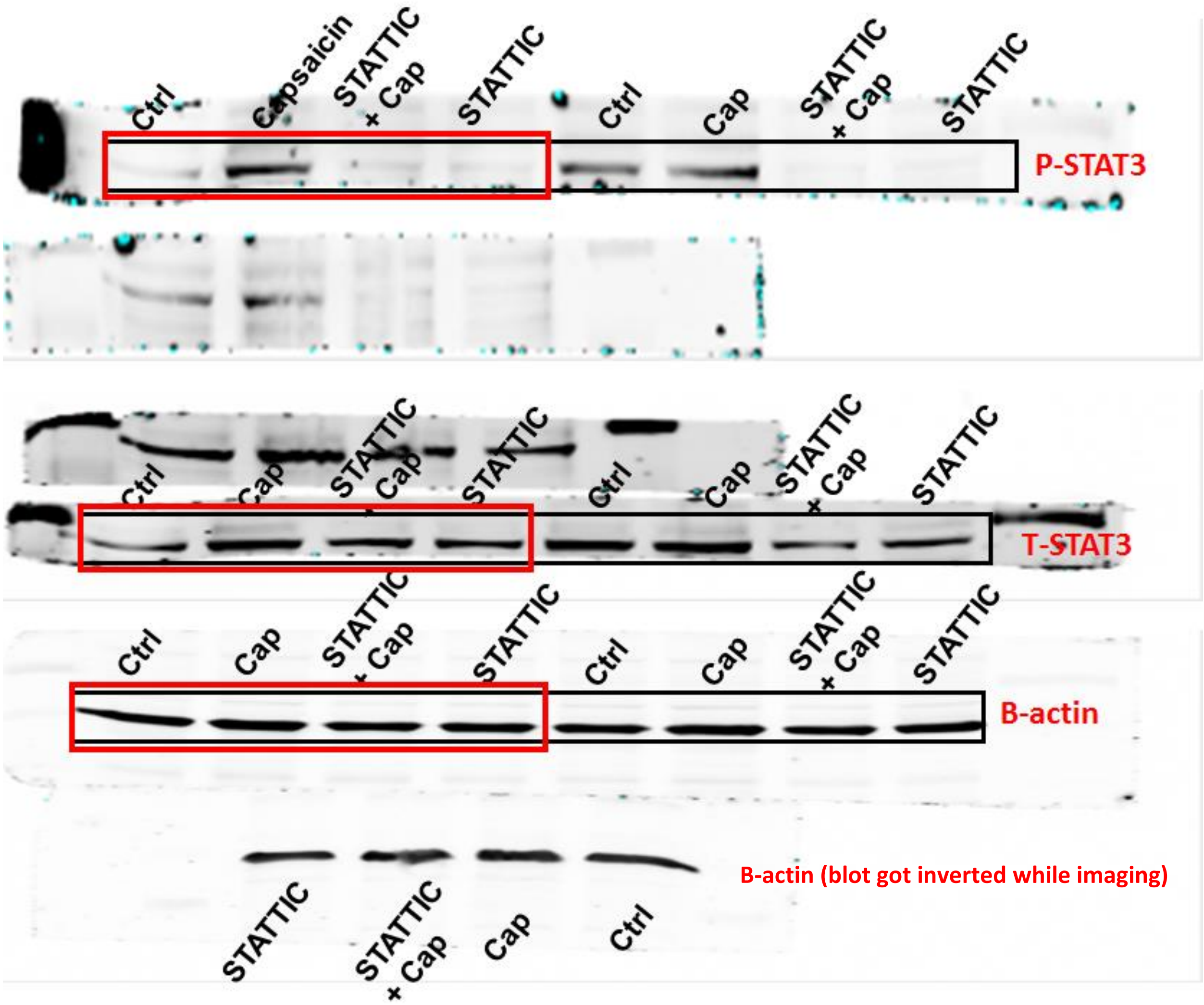

Figure 4C: Capsaicin induced STAT1 phosphorylation is TRPV1 dependent

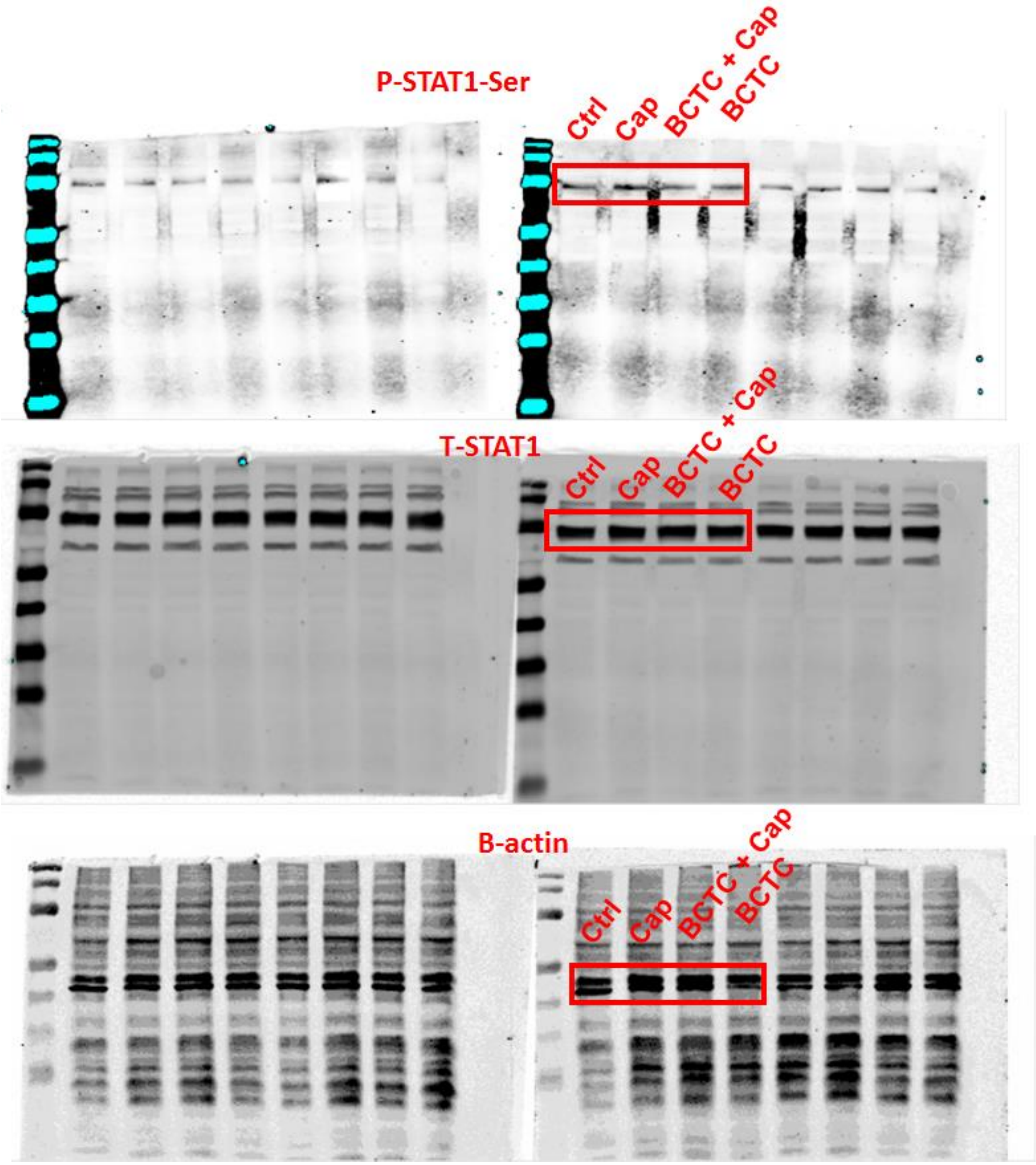

Figure 4D: Capsaicin induced STAT3 phosphorylation is TRPV1 independent

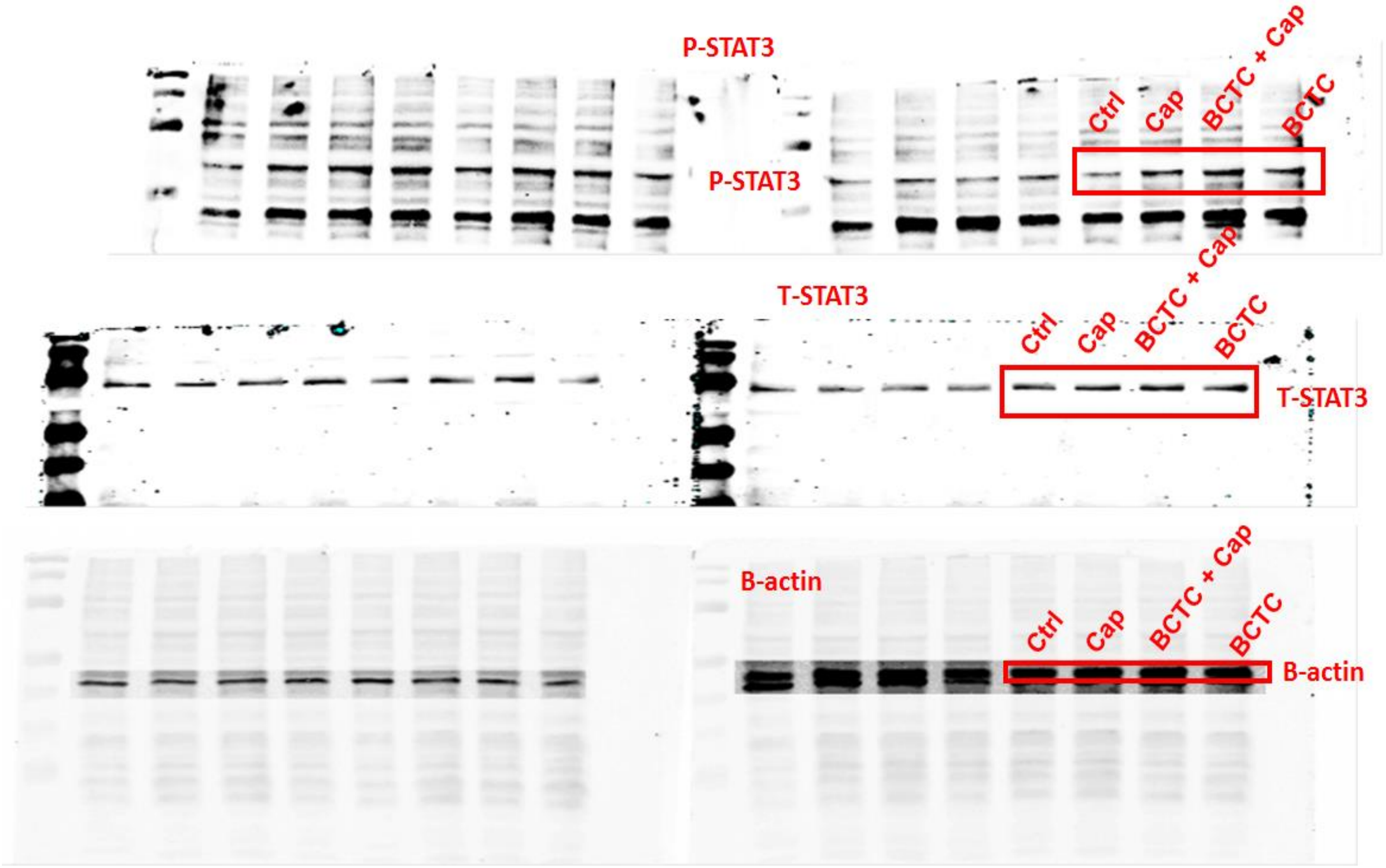

Different exposure of b-actin for 4D

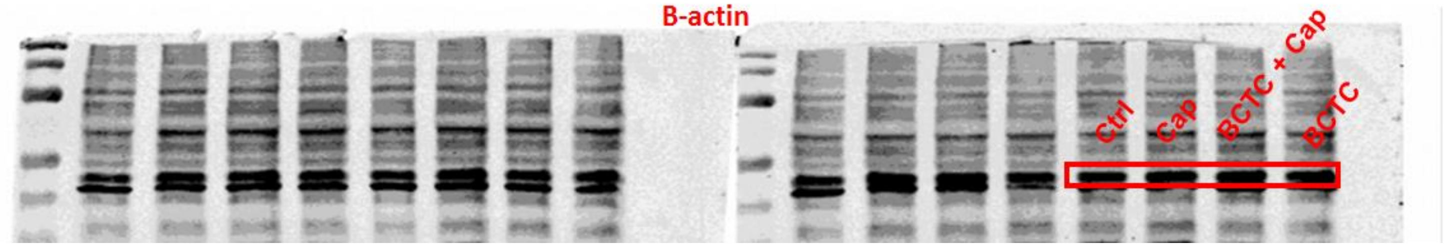

Figure 5A: Capsaicin induced STAT1 phosphorylation is CB1 independent

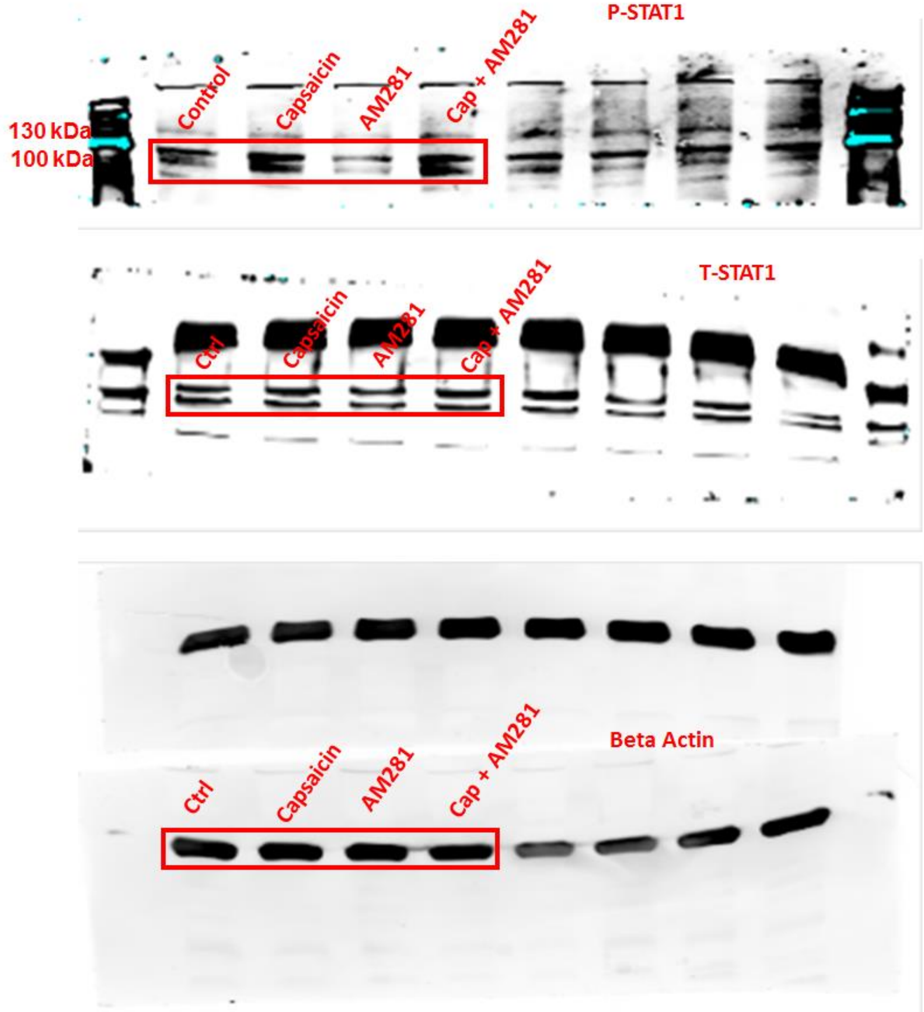

Fig. 5B: Capsaicin induced STAT3 phosphorylation is CB1 independent

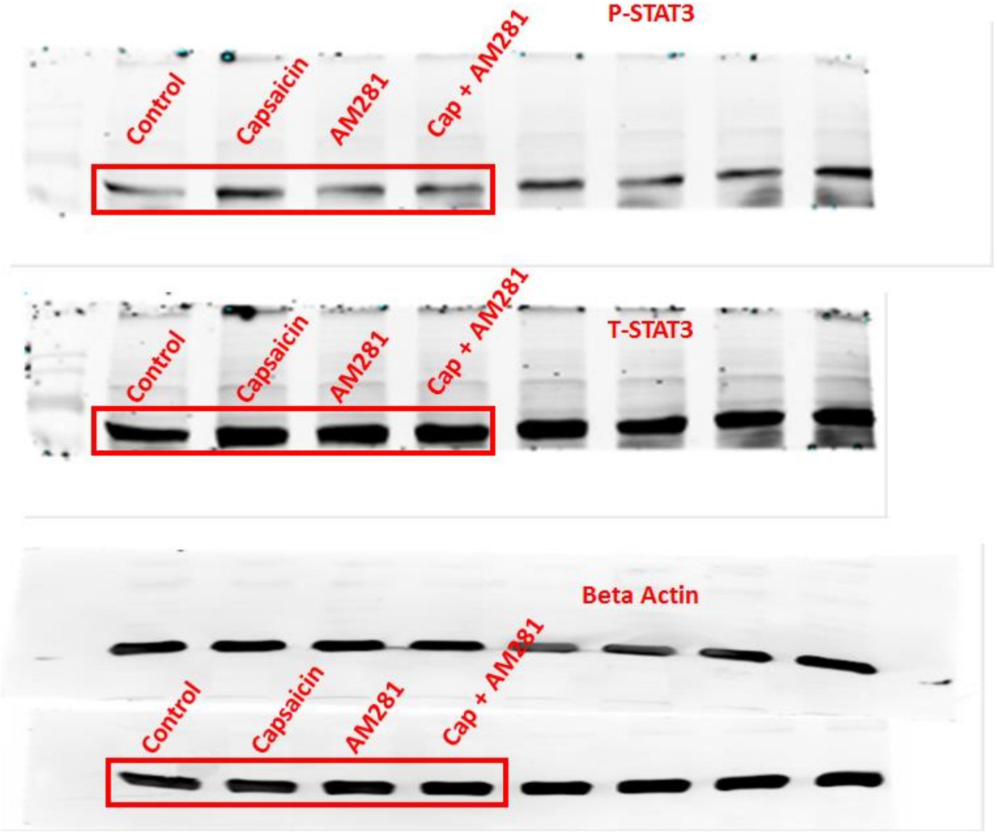

Figure 5C: Capsaicin induced STAT1 phosphorylation is CB2 dependent

- Beta actin is used for loading control. Unfortunately, the file was corrupted.

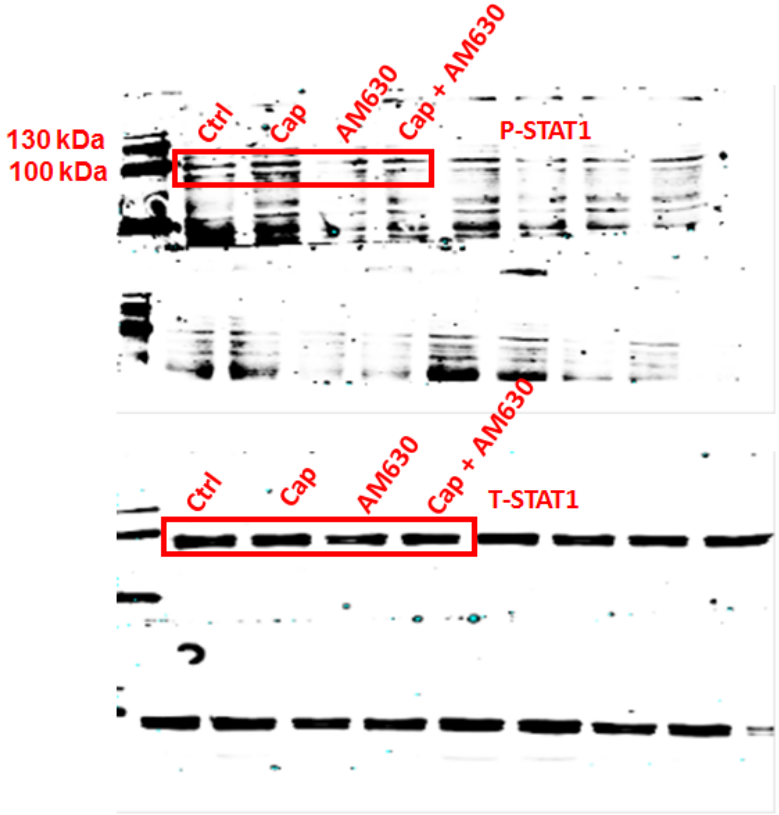

Fig. 5D: Capsaicin induced STAT3 phosphorylation is CB2 dependent

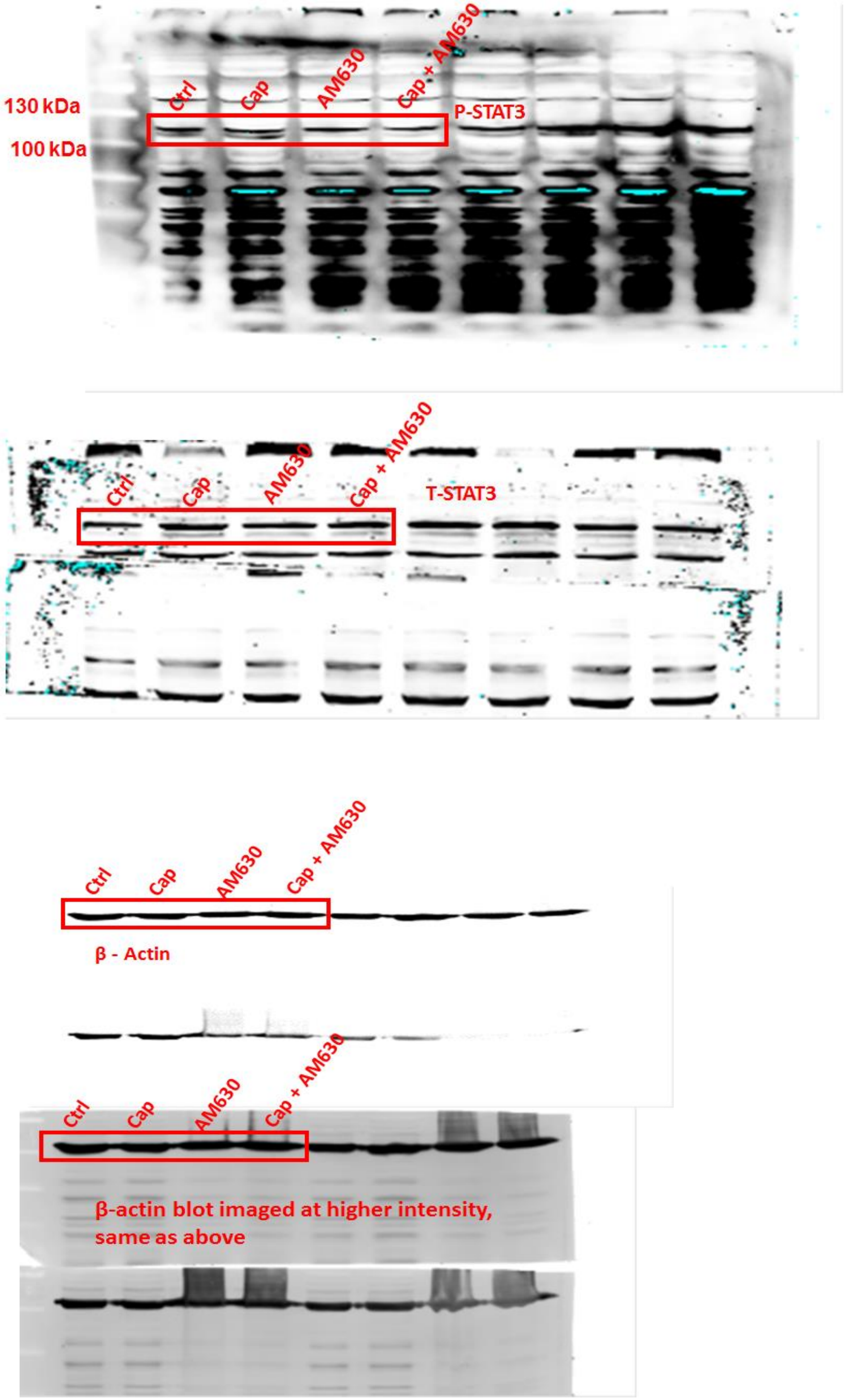

Figure 5E: CB2 by JWH 015 activation induces STAT3 phosphorylation

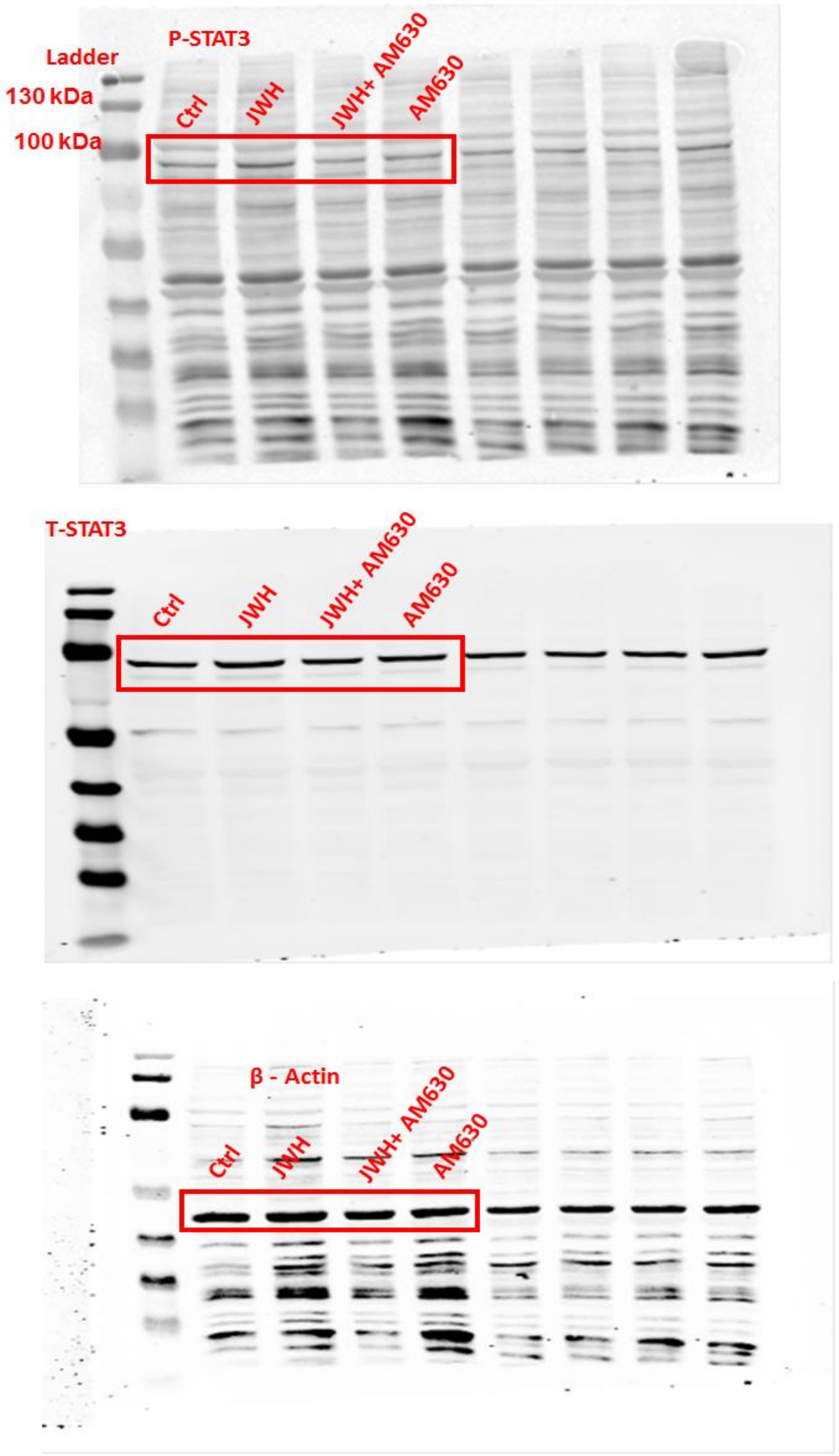

Supplementary Figure S1A: Capsaicin increases STAT1 phosphorylation at 45 - 60 minutes

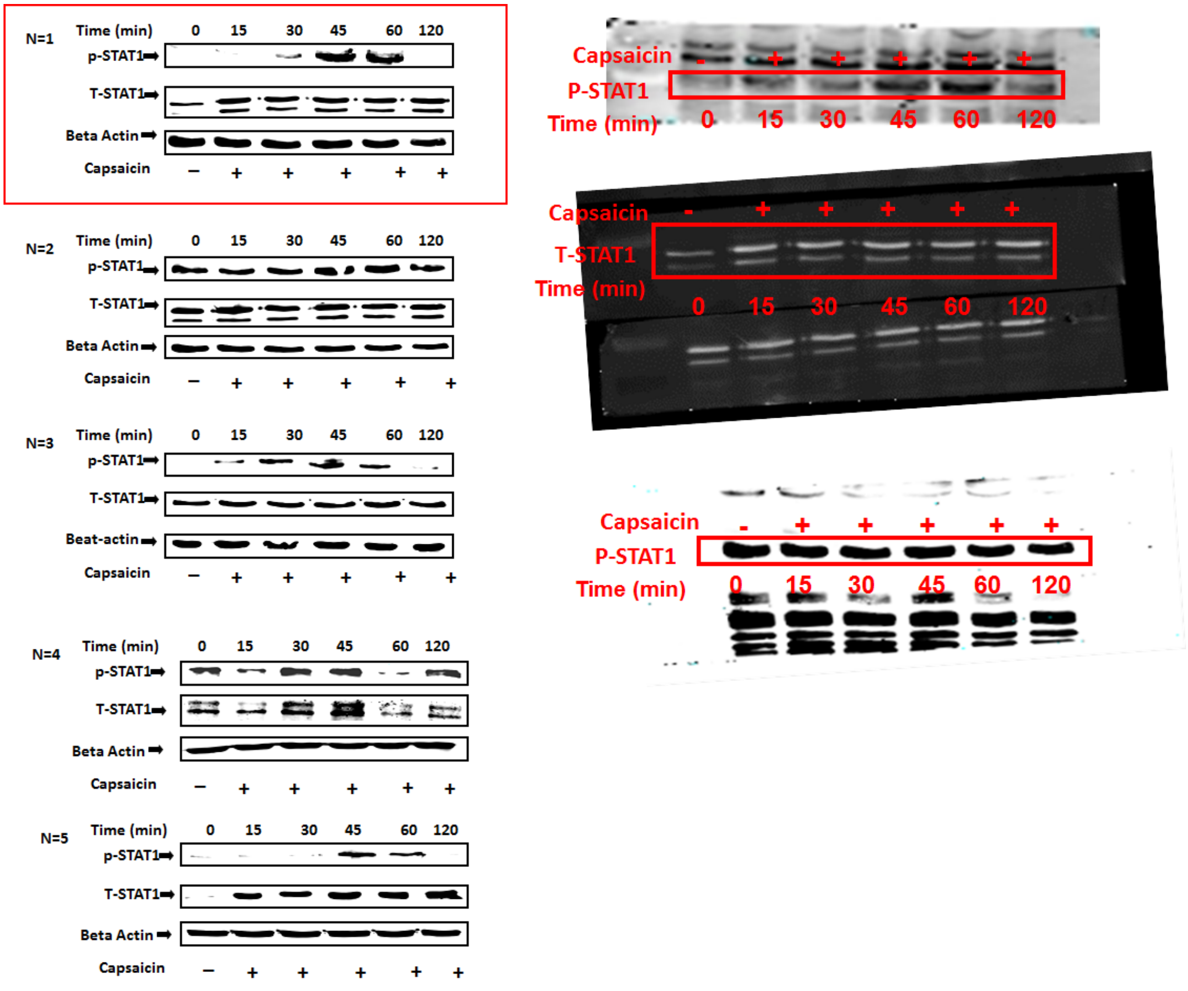

Supplementary Figure S1B: Cisplatin increases STAT1 phosphorylation

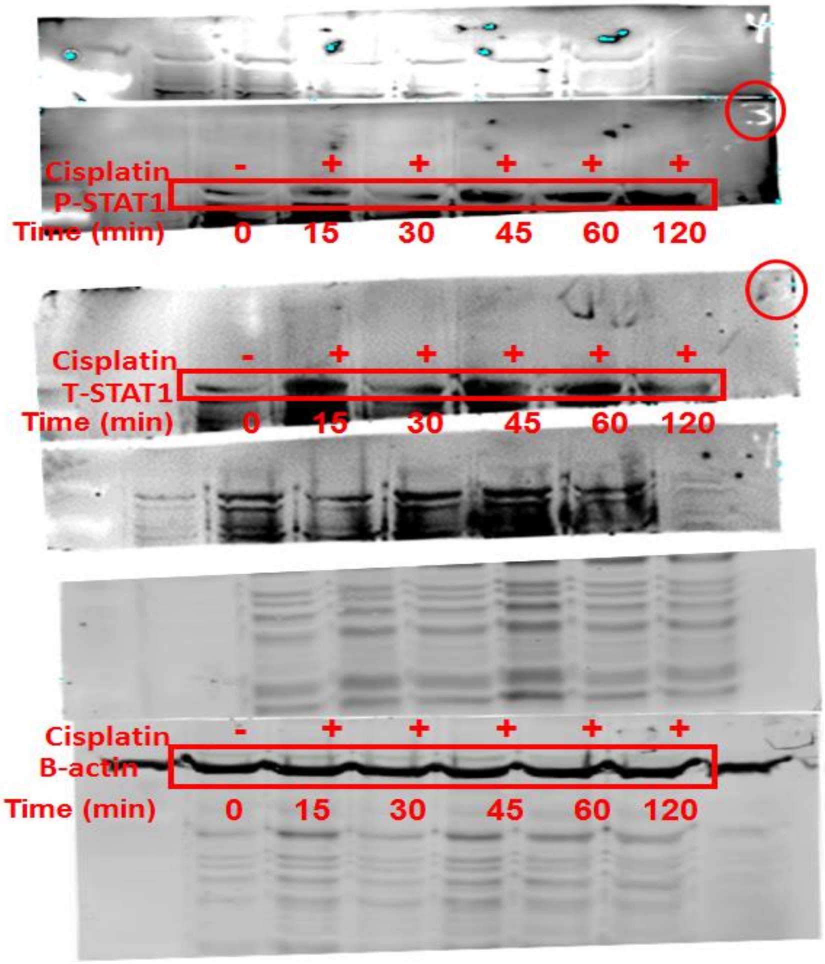

Supplementary Figure S2A: Capsaicin increases JAK2

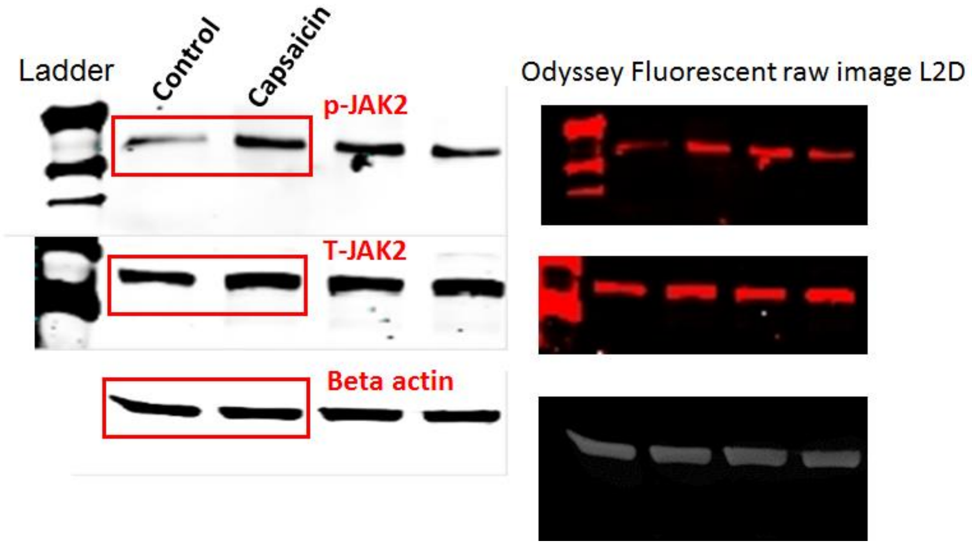

Supplementary Figure S2B: Cisplatin decreases JAK2 phosphorylation

- The original blots are unavailable as the file is corrupted.

Supplementary Figure S3A: Capsaicin increases CB2

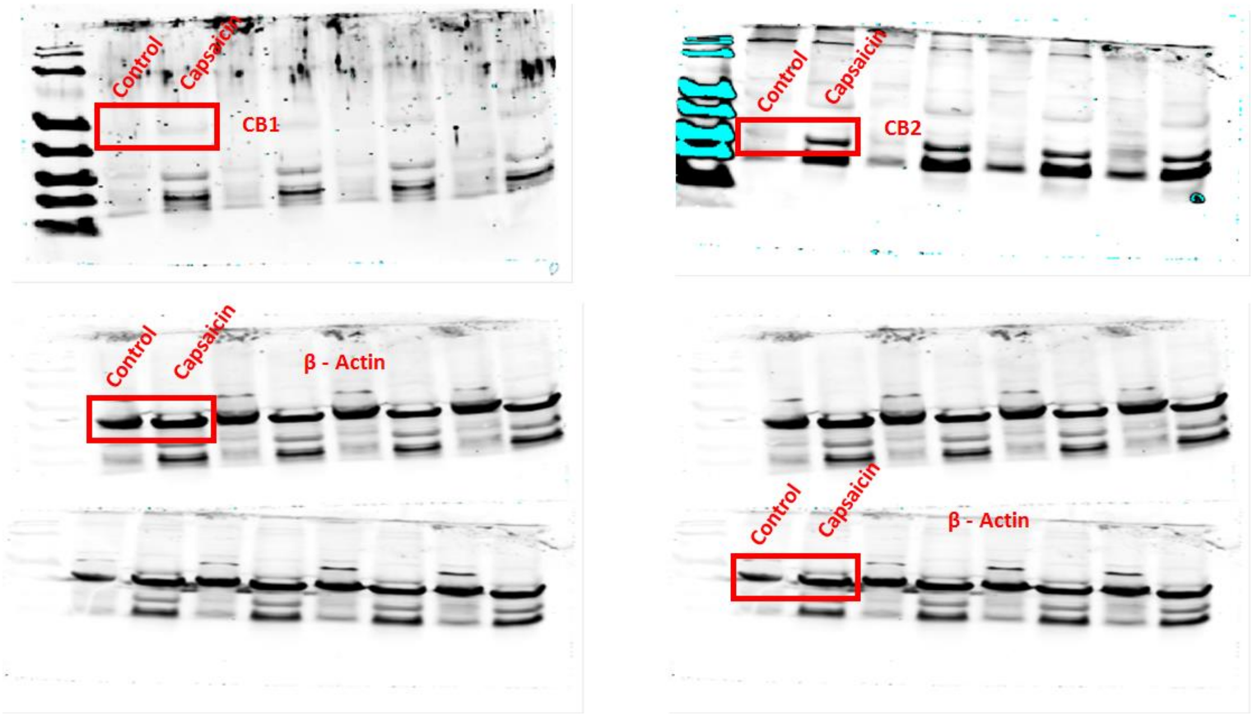

Supplement: Supplementary file 1 — Supplementary Figures [file 41598_2019_40425_MOESM1_ESM.pdf]
